# Mechanical stimulation and electrophysiological monitoring at subcellular resolution reveals differential mechanosensation of neurons within networks

**DOI:** 10.1038/s41565-024-01609-1

**Published:** 2024-02-20

**Authors:** Krishna Chaitanya Kasuba, Alessio Paolo Buccino, Julian Bartram, Benjamin M. Gaub, Felix J. Fauser, Silvia Ronchi, Sreedhar Saseendran Kumar, Sydney Geissler, Michele M. Nava, Andreas Hierlemann, Daniel J. Müller

**Affiliations:** 1 Department of Biosystems Science and Engineering, ETH Zurich, Basel, Switzerland; 2 Allen Brain Institute, Seattle, WA USA; 3 Maxwell Biosystems, Zurich, Switzerland

**Keywords:** Nanobiotechnology, Nanoscale devices, Bionanoelectronics, Characterization and analytical techniques

## Abstract

A growing consensus that the brain is a mechanosensitive organ is driving the need for tools that mechanically stimulate and simultaneously record the electrophysiological response of neurons within neuronal networks. Here we introduce a synchronized combination of atomic force microscopy, high-density microelectrode array and fluorescence microscopy to monitor neuronal networks and to mechanically characterize and stimulate individual neurons at piconewton force sensitivity and nanometre precision while monitoring their electrophysiological activity at subcellular spatial and millisecond temporal resolution. No correlation is found between mechanical stiffness and electrophysiological activity of neuronal compartments. Furthermore, spontaneously active neurons show exceptional functional resilience to static mechanical compression of their soma. However, application of fast transient (∼500 ms) mechanical stimuli to the neuronal soma can evoke action potentials, which depend on the anchoring of neuronal membrane and actin cytoskeleton. Neurons show higher responsivity, including bursts of action potentials, to slower transient mechanical stimuli (∼60 s). Moreover, transient and repetitive application of the same compression modulates the neuronal firing rate. Seemingly, neuronal networks can differentiate and respond to specific characteristics of mechanical stimulation. Ultimately, the developed multiparametric tool opens the door to explore manifold nanomechanobiological responses of neuronal systems and new ways of mechanical control.

## Abstract (web summary)

Here, the authors combined and synchronized single-cell nanoindentation, electrophysiology and functional fluorescence imaging to evaluate the responses of neuronal networks to mechanical stimuli with piconewton force sensitivity and nanometre precision, enabling the exploration of nanomechanobiology and manipulation of neuronal systems.

## Main

Within complex tissue, mechanical cues interact with cellular compartments featuring diverse functional properties and elicit various cellular responses. For example, changes in plasma membrane tension can modulate cell polarity and migration^1,2^. Alterations in the viscoelastic properties of cytoskeletal networks, formed by various proteins^3,4^, play key roles in substrate sensing^5^, cell division^6^, cell migration, chemotaxis and other processes^7,8^. Strain-induced nucleus deformations help cells to measure and respond to spatial constraints^9^. Neurons are no exception to this. In addition, neurons feature electrophysiologically distinguishable compartments, including the soma, dendrite and axon, which have very different rheological properties^10^. For example, the soma behaves like a soft elastic solid, whereas neurites are stiffer and more viscous. Such distinct mechanical properties of neuronal compartments entail distinct functional responses to mechanical signals^11^. In the context of substrate stiffness and neurite migration, neurons grown on stiffer substrates show longer axons^12^. In vivo, sensing and exposure to mechanical cues are important, especially in the mid-diencephalon, where the optical tract undergoes caudal bending that coincides with steep stiffness gradients in the tissue^12^. Similarly, during learning, expanding spines of potentiated excitatory glutamatergic synapses can push the axonal boutons, forcing an actin-polymerization-dependent SNARE (soluble *N*-ethylmalemeide-sensitive factor attachment protein receptor) complex assembly and neurotransmitter release^13^, thus suggesting a synaptic pressure sensation and transmission model. In the context of neuromodulation, externally applied mechanical forces can induce calcium spikes in neurons^11^. In mouse brain slices, pyramidal neurons in the neocortex and hippocampus are mechanosensitive^14^. Concurrently, ultrasound neuromodulation has been intensively explored to modulate neuronal activity in the central nervous system^15–18^. This growing evidence highlights the direct and intricate role of mechanical cues in modulating neuronal activity. However, to better understand which mechanical properties neurons exhibit, how neurons sense and respond to mechanical cues, and how this mechanical information reflects in neuronal activity, that is, the corresponding trains of action potentials, requires new multiparametric tools.

Among currently available techniques to study the mechanobiology of neuronal networks, the synchronization of shear-stress delivering pistons or atomic force microscopy (AFM) with functional fluorescence imaging (for example, GCaMP6s) cannot provide the precise spike times of action potentials^11^. Genetically encoded calcium sensors from the GCaMP family are often used to monitor neuronal activity^19^. However, intricate calcium signalling inside cells implies that readouts of calcium sensors may not fully capture neuronal action potentials in response to mechanical stimuli. Furthermore, combinations with voltage imaging (for example, genetically encoded voltage indicators) provide spike times, although the physical motion of the cell membrane can render voltage imaging techniques incapable of providing information on the waveform features of the spikes^20^. Combining AFM or nanoindenters with patch clamp provides waveform features and precise times of the spike at one neuronal location (typically the soma)^21,22^. However, these tools cannot assess the subcellular electrophysiology of neuronal networks, and long-term mechanical measurements on patched neurons entail limited cell viability and altered neuromechanical properties caused by patch-clamping^23^. Furthermore, previously reported combinations of AFM or nanoindenters with opaque multielectrode arrays (MEAs) are not compatible with optical microscopy and subcellular resolution, which are required to locate, mechanically manipulate and electrophysiologically monitor individual neurons and neurites^24^.

To address these shortcomings, we here combine and synchronize AFM, long-working-distance optical microscopy and a high-density (HD)-MEA to perform functional fluorescence imaging and visualize electrophysiological dynamics simultaneously on hundreds of rat cortical neurons at subcellular resolution while measuring their mechanical properties and applying mechanical stimuli at nanometre precision, piconewton force resolution and millisecond time resolution.

## Mechanobiological characterization of neuronal systems

**Fig. 1:**
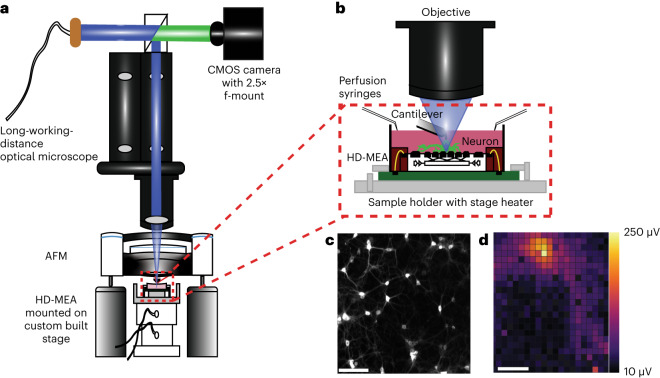
Schematic, working principle and workflow of the mechanoelectrophysiological assay. **a**, Alignment of optical microscope, AFM and HD-MEA. The alignment ensured that the fluorescence, emitted by the sample on the HD-MEA, was collected through a sapphire window in the cantilever holder of the AFM and passed through the optical microscope to a large CMOS sensor camera via a 2.5× f-mounted projector lens. The optical microscope is equipped with a TTL-pulse-controllable monochromatic LED illuminator for filter-cube-free fluorescence imaging. Fluorescence images of the entire chip are used to correlate the locations of the neurons with electrodes of the HD-MEA chip. The HD-MEA is mounted on a custom-made, piezo-controlled *x*,*y* stage, while the AFM moves the AFM cantilever in the *z* direction. **b**, Zoom-in of the red dashed box in **a** showing the AFM cantilever positioned above a neuron. A stage heater and perfusion set-up enable temperature and nutrient control. **c**, Fluorescence image of rat cortical neurons seeded on the HD-MEA and labelled with membrane stain (NeuO). **d**, Electrophysiological footprint of a neuron acquired by the HD-MEA. Scale bars, 100 µm.

HD-MEA chips with 26,400 electrodes (17.5 µm pitch) were used to monitor extracellular potentials at subcellular resolution from neuronal networks^25^. The chips were mounted on a custom-made *x*,*y* stage and sample holder, complemented with a stage heater and syringes for media perfusion (Fig. 1a,b and Supplementary Fig. 1). The AFM head and HD-MEA stage were aligned with the optical path of a long-working-distance fluorescence microscope to visualize at ∼40× magnification single neurons and neurites cultured on HD-MEA chips (Fig. 1c and Supplementary Video 1). AFM, camera and HD-MEA were transistor–transistor logic (TTL)-pulse-synchronized using a high-speed optocoupler to provide precise time stamps on the mechanical, optical and electrophysiological data. Fluorescence images of neurons were obtained across the entire chip and correlated with electrode numbers (Supplementary Fig. 2). While the AFM head remained stationary and aligned with the optical path, the HD-MEA chip was moved to place the neuronal compartment of interest under the AFM cantilever for mechanical characterization and manipulation. Finally, 1,024 HD-MEA electrodes were routed to simultaneously record extracellular potentials around and under the neurons of interest at 20 kHz sampling frequency and 300 Hz cut-off high-pass filter. An unsupervised template-matching spike sorter obtained electrical ‘footprints’ that represent extracellular electrical potential landscapes and spike times of single neurons^26^ (Fig. 1d). Calcium imaging was used for the immediate visual identification of neuronal activity.

**Fig. 2:**
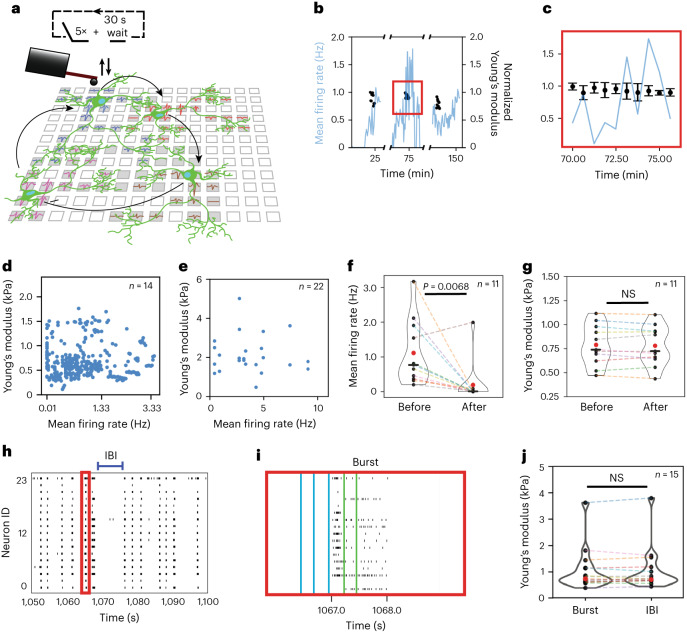
Correlated stiffness and electrophysiology measurements of neuronal networks. **a**, Schematic and protocol for correlating the mechanical stiffness of neuronal somas and the firing rate within neuronal networks over several hours. Neurons are shown in green, squares represent HD-MEA electrodes, and grey filled squares represent user-defined electrodes routed to recording channels. Recorded extracellular waveforms of the neurons are given as coloured traces (blue, red, brown and magenta). **b**, Time series of mean firing rates of a single neuron (blue lines) and Young’s modulus of the neuronal soma (black dots). Each black dot represents the average of at least three stiffness measurements. **c**, Zoom-in of the red box in **b**. Error bars show s.d. **d**, Scatter plot of mean firing rates from *n* = 14 individual neurons versus Young’s modulus of their neuronal somas. A linear Pearson correlation test between firing rates and stiffness revealed no strong correlation (*r* = 0.03, *P* = 0.56). **e**, Scatter plot of the mean firing rates of neurons and Young’s modulus of *n* = 22 neurites measured from *n* = 11 individual neurons. **f**,**g**, Mean firing rate (**f**) and Young’s modulus (**g**) of *n* = 11 individual neurons before and after addition of glutamate receptor antagonists 10 μM DNQX and 40 μM D-AP5. **h**, Raster plot showing the synchronized spiking activity (action potentials) of neurons, with bursts (red box) and IBIs marked. **i**, Zoom-in of the red box in **h**, showing a burst of spikes of the neuronal network. Blue lines indicate the time points at which the force–displacement curves were collected to approximate the Young’s modulus of the soma of a neuron during IBIs; green lines indicate the time points to approximate the Young’s modulus of a neuron during a burst. **j**, Young’s modulus of *n* = 15 individual neuronal somas during bursting and IBIs. In all violin plots red dots represent mean values, black dots data from single neurons, and black lines the median of the population. Wilcoxon signed-rank test was used to determine statistical significance. *P* values are given in figures, with values >0.05 being considered non-significant (NS). Source data

Actin filaments and other cytoskeletal components that contribute to the mechanical properties of neurons interact with voltage-gated Na^+^ and K^+^ channels^27^ and subunits of AMPA (α-amino-3-hydroxy-5-methyl-4-isoxazole propionic acid) and NMDA (*N*-methyl-d-asparticacid) receptors^28^. Generally, these cytoskeletal components help cells to adapt to the viscoelastic properties of their surroundings^29^. Studies report that stiff substrates enhance the activity of cultured cortical neurons^30^. Hence, we monitored alterations in firing rate and the stiffness of neurons for several hours to find possible correlations (Fig. 2a). Briefly, every 30 s we recorded the average stiffness of a neuronal soma through five AFM force–displacement curves for up to 5 min, and then characterized the next neuron. For stiffness measurements we used a 5-µm-diameter bead glued to the free end of the cantilever. After 1 h, we returned to the same set of neurons and repeated the stiffness measurements for 5 min. This cycle was repeated three times to track the stiffness of each soma for up to 3 h. Throughout the stiffness measurements, we monitored the electrical activity of the neuronal network and extracted the mean firing rates of each neuron (Fig. 2b–d). No strong correlations between the stiffness of the soma and the mean neuronal firing rate were observed. To further address whether the stiffness of neurites correlates with the firing rate, the firing activity of sparsely distributed rat cortical neurons was recorded for 5 min and, thereafter, the stiffness of two basal neurites measured. No correlations were found between the stiffness of the neurites and the neuronal firing rate (Fig. 2e). Finally, the stiffness of the neuronal soma was measured before and after suppression of excitatory synaptic connections through the glutamate receptor antagonists DNQX (6,7-dinitroquinoxaline-2,3-dione) and D-AP5 (d-2-amino-5-phosphonopentanoic acid) (Fig. 2f,g). Although the firing rate decreased considerably, no differences in the stiffness of the soma were observed.

Cultured rat cortical neurons developed synchronous spontaneous activity after 20 days in vitro (DIV), which was electrophysiologically recorded as bursting events of the neuronal network (Fig. 2h). During bursts, the neuronal firing frequency massively increased for short periods, whereas the time between bursts was denoted as the ‘inter-burst interval’ (IBI) (Fig. 2i). The impact of rapid changes in the mean firing rate was explored by extracting the stiffness of neuronal somas from force–displacement curves collected during bursts and IBIs (Fig. 2j). The analysis showed that the soma did not change stiffness between bursting period and IBI.

## Mechanically evoked action potentials

Depending on the subcellular location and magnitude, mechanical stimuli can evoke calcium responses in cortical and hippocampal neurons^11,31^, which range in duration from a few (*τ* = 3.4 s) to several (*τ* = 24.1 s) seconds^11^. The presence of mechanically gated ion channels in pyramidal neurons of the neocortex and hippocampus was reported previously^14^. However, information on the action potentials generated in cortical neurons in response to mechanical stimulation is lacking. Thus, we decided to mechanically stimulate rat primary cortical neurons while recording their calcium response and electrophysiological footprints.

**Fig. 3:**
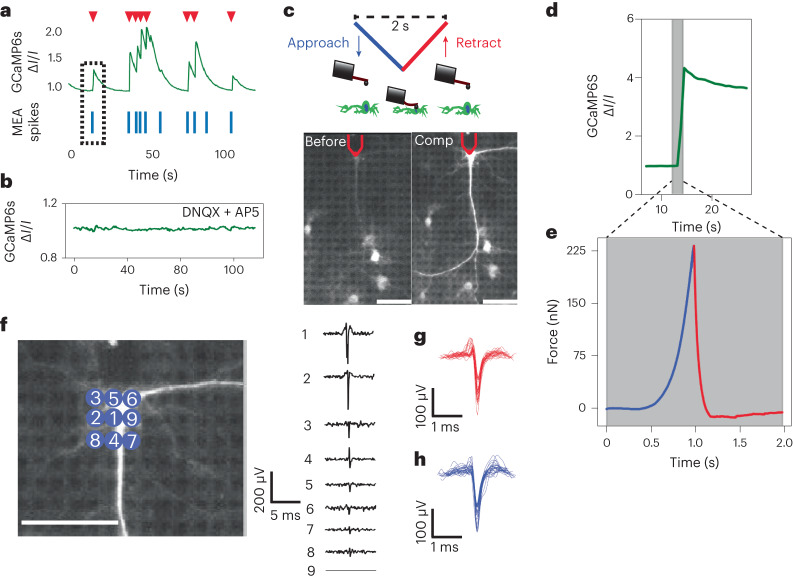
Transient mechanical stimulation evokes action potentials. **a**, Fluorescence intensity (green line) of a spontaneously firing neuron expressing the fluorescent calcium reporter GCaMP6s. Blue lines show spike times of the same neuron recorded by the HD-MEA. Red triangles highlight the GCaMP6 fluorescence peaks. The black dotted box shows the alignment of calcium responses and HD-MEA signals. **b**, Fluorescence intensity (green line) of a neuron expressing GCaMP6s after adding the synaptic-activity blockers DNQX and D-AP5. **c**, Cartoon showing the mechanical stimulation of a neuron (top panel). Fluorescence images of the rat cortical neuron on the HD-MEA chip expressing GCaMP6s before (left) and compression (right) mechanical stimulation (bottom panel). Red lines outline the free end of the AFM cantilever with the 5 µm bead. Scale bars, 105 µm. **d**, Normalized fluorescence intensity of mechanically stimulated neurons expressing GCaMP6s. The grey window shows the stimulation period. **e**, A force–time curve recorded during mechanical stimulation of the neuron. Approach (blue) and retraction (red) of the bead are shown in **c**. **f**, Left, fluorescence image of a stimulated neuron with HD-MEA electrodes indicated with different numbers. Right, extracellular potentials detected on the corresponding electrodes (numbers) during mechanical stimulation. Scale bar, 105 µm. **g**,**h**, Superimposed waveforms of mechanically evoked (**g**) and spontaneously occurring (**h**) action potentials (*n*_spikes_ = 50 obtained from 16 independent neurons). Comparing the amplitudes and halfwidth of the mechanically evoked (**g**) and spontaneously occurring action potentials (**h**) with a Mann–Whitney *U-*test resulted in *P* values of 0.098 and 0.11, respectively. Source data

To block the spontaneous activity of neurons that can mask mechanically evoked responses, glutamate receptor antagonists were added (Fig. 3a,b). Thereafter, we applied transient subtraumatic pressures to evoke calcium responses in neurons^11^. Briefly, the 5-µm-diameter bead, glued to the cantilever, was indented on the soma of a neuron until detection of a setpoint force of ∼200 nN (∼5 kPa) and then immediately (∼10 ms) retracted (Fig. 3c). Upon reaching the setpoint force, the neuron showed a calcium response, which lasted for several seconds (*τ* = 26.2 s), along with a depolarization event detected on several electrodes (Fig. 3d–g). This transient (∼500 ms) mechanical stimulation of the soma evoked the neuron to depolarize with a latency of 130.17 ms (s.d., 217.61 ms) (Supplementary Fig. 3). The electrodes detected a mean maximum of the mechanically evoked spike amplitude of 222.76 µV (s.d., 68.81) and full-width-at-half-maximum of 0.31 ms (s.d., 0.04 ms), which were comparable to the spontaneously generated mean spike amplitude of 188.12 µV (s.d., 47.97 µV) and full-width-at-half-maximum of 0.34 ms (s.d., 0.06 ms) (Fig. 3h).

**Fig. 4:**
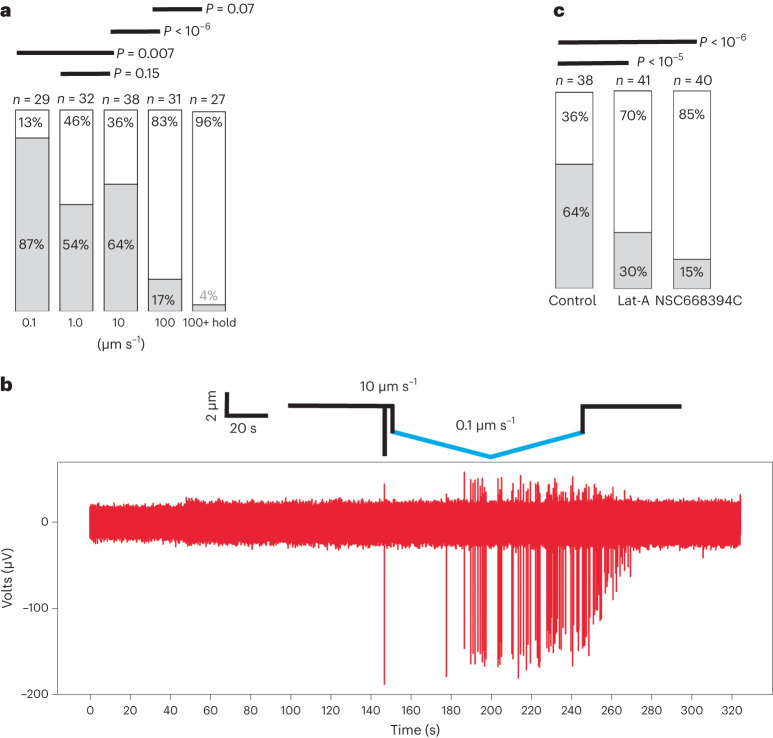
Influence of the indentation speed and anchoring between neuronal membrane and actin cytoskeleton on mechanically evoked action potentials. **a**, Bar plots depict the percentage of cortical neurons showing a response (grey) and no response (white) to mechanical stimuli of a bead indenting the soma at various indentation speeds. The neurons were indented with a 5-μm-diameter silica bead, glued to the AFM cantilever, by applying a force of 200 nN (∼5 kPa). The response of the neurons expressing the genetically encoded calcium sensor GcaMP6s was monitored by confocal microscopy. **b**, Differential neuronal responses to indentations at different speeds. Time series of extracellular potentials of a cortical neuron, in which soma was indented with a 5 µm bead. The solid line at the top represents the vertical movement of AFM cantilever and bead at 10 µm s^–1^ (black) and 0.1 µm s^–1^ (blue). Number of action potentials evoked through 10 µm s^–1^ and 0.1 µm s^–1^ indentations were compared using a two-tailed Mann–Whitney *U*-test. The *P* value of 0.004 (*n* = 8 individual neurons) suggests that neurons can differentiate indentation speeds. **c**, Coupling between the neuronal membrane and the actin cytoskeleton is required for cortical neurons to respond to mechanical stimuli. Bar plots give the percentage of cortical neurons showing a response (grey) and no response (white) to mechanical stimulation of untreated (control) neurons and of neurons treated with 0.1 μM latrunculin A (LatA) or 50 μM NSC668394C. The neurons were indented with a 5-μm-diameter silica bead by applying a force of 200 nN (∼5 kPa) at 10 μm s^–1^. The neurons expressed the genetically encoded calcium sensor GCaMP6s. The response of the neurons expressing GCaMP6s was monitored by confocal microscopy. In all experiments, spontaneous activity of the neurons was blocked with glutamate receptor antagonists (10 μM DNQX and 40 μM D-AP5) to isolate mechanically evoked responses. *n* represents the number of characterized independent neurons. *P* values of the *χ*^2^ analysis for different conditions are given above the bars in **a** and **c**. Source data

To test whether the indentation speed affects the mechanically evoked action potentials of cortical neurons, we indented somas at ∼5 kPa, with four different speeds: 0.1, 1.0, 10.0 or 100 µm s^–1^ (Fig. 4a). To better monitor morphological changes, AFM was combined with confocal microscopy to stimulate neurons seeded on glass coverslips^11^, and monitor their responses through GCaMP6s (Supplementary Fig. 4 and Supplementary Video 2). To isolate mechanically evoked responses of neurons, their spontaneous activity was blocked with glutamate receptor antagonists. Most cortical neurons (87%) responded to mechanical indentation at 0.1 µm s^–1^, 54% at 1 µm s^–1^, 64% at 10 µm s^–1^ and only 17% at 100 µm s^–1^. The duration for indenting a neuron at the slowest speed of 0.1 μm s^–1^ approaches 60 s (Supplementary Fig. 5). To learn whether the different neuronal responses to mechanical indentions resulted from the indentation speed or duration, we indented the neuronal soma with 100 μm s^–1^ and kept the bead indenting the soma for 60 s before retracting the cantilever (Fig. 4a, ‘100+ hold’). Only 4% of the cortical neurons responded, thus suggesting that the speed and not the duration of the indentation caused cortical neurons to respond differently.

As the indentation at 0.1 µm s^–1^ yielded the highest rate of neuronal responses, we chose this indentation speed to investigate further the nature of the neuronal action potentials using the AFM–HD-MEA device (Fig. 4b). Cortical neurons, indented at 0.1 µm s^−1^, responded with multiple action potentials, whereas only single action potentials were evoked at 10 µm s^–1^. To test whether indenting at different speeds entails varying threshold forces for mechanically evoked responses, cortical neurons were indented by increasing forces from 10 to 400 nN (∼0.25–10 kPa). Indeed, the threshold forces evoking neuronal responses were lower at 0.1 µm s^−1^ (mean, 182.75 nN; s.d., 84.86 nN) than at 10 µm s^−1^ (mean, 264.31 nN; s.d., 74.28 nN) (Supplementary Fig. 6). These findings demonstrate that neurons respond more sensitively to mechanical stimuli at lower speed.

Next, the actin cytoskeleton and its anchoring to the neuronal membrane were perturbed to understand their role in mediating mechanically evoked responses of cortical neurons. Most neurons (∼70%), pretreated with latrunculin A, which depolymerizes actin filaments, did not respond to mechanical stimulation (Fig. 4c and Supplementary Fig. 7). Furthermore, most neurons (∼85%) treated with NSC668394C, which prevents the phosphorylation of ezrin^32^, the subsequent binding of ezrin to actin, and, thus, the anchoring of the neuronal membrane to the actin cytoskeleton^33–36^, did not respond to mechanical stimuli either, while the actin cytoskeleton remained unperturbed. The viability of neurons in the presence of latrunculin A or NSC668394C was confirmed by time-lapse confocal microscopy (Supplementary Fig. 8).

## Effect of compression on spontaneous neuronal activity

**Fig. 5:**
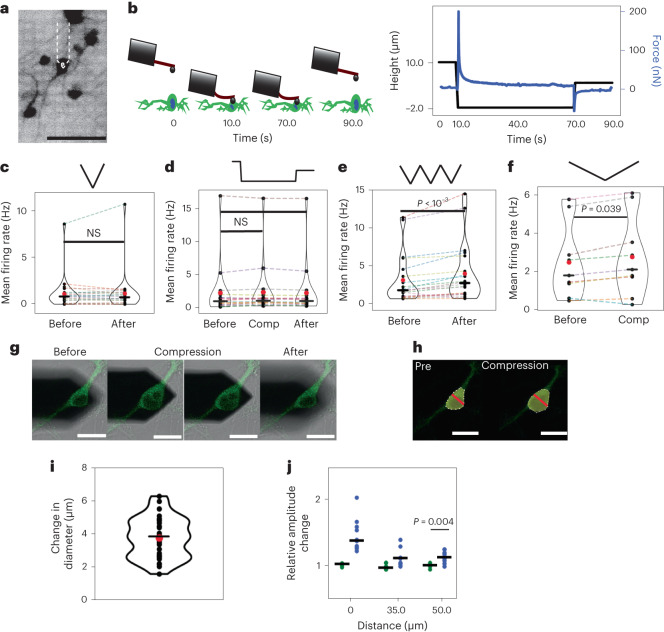
Effects of transient and static mechanical compression on spontaneous activity of neurons. **a**, Fluorescence image of membrane-stained (NeuO) rat cortical neurons on the HD-MEA chip. The white dashed lines outline the AFM cantilever and the white circle marks the 5 µm bead. Scale bar, 105 µm. **b**, Force–time curve collected while compressing the neuronal soma for 60 s, thus applying static compression. The black line shows the movement of the cantilever; the blue line shows the force response of the soma during compression. **c**, Mean firing rate of neurons before and after single transient compression with 5 kPa (*n* = 22 individual neurons, *n*_spikes_ > 2,000) at an indentation speed of 10 µm s^–1^. **d**, Mean firing rate of neurons 2.5 min before, during and 2.5 min after a 60 s static compression with ∼5 kPa (*n* = 17 individual neurons, *n*_spikes_ > 2,000). **e**, Mean firing rate of neurons 2.5 min before and 2.5 min after three consecutive transient compressions with ∼5 kPa (*n* = 20 individual neurons, *n*_spikes_ > 2,000). **f**, Mean firing rate of neurons before and during one transient compression with ∼5 kPa (*n* = 8 individual neurons, *n*_spikes_ > 2,000) at an indentation speed of 0.1 µm s^–1^. **g**, Confocal fluorescence images of a neuron showing morphological changes of the soma during 60 s static compression of ∼5 kPa. Scale bars, 20 µm. **h**, Confocal fluorescence images of a neuron fitted with *n*-sided polygons to assess the changing diameter (red line) of the soma. The diagonal showing the largest diameter change during compression was used for measurement. Scale bars, 20 µm. **i**, Changing diameter of soma during compression. Changes represent the difference in the soma diameter before and after compression (*n* = 30 individual neurons). **j**, Relative changes in amplitude of the action potentials as a function of distance of an electrode from the compression site (target electrode) for control (green dots, *n* = 10 individual neurons) and 5 kPa (blue dots, *n* = 10 individual neurons). Black dots represent individual neurons, red dots mean values and black lines median values in **c–f**, **i** and **j**. A Wilcoxon signed-rank test was used in **c**–**f** and **j**. *P* values are given in the figures; *P* > 0.05 is considered non-significant. Source data

Spontaneous activity of neurons includes sensory-input-independent firing in in vivo and in vitro neuronal cultures through active synaptic connections^37^. Although shear forces or focused ultrasound can mechanically stimulate responses of hundreds of neurons within spontaneously active neuronal networks^11,38^, it remains unclear how single neurons react to localized mechanical forces or pressures and adapt their firing pattern. Thus, the neuronal soma with active synaptic connections was subjected to transient and static compressions. For transient compression the 5 µm bead was lowered on the neuronal soma at 0.1 or 10 µm s^–1^ until reaching the setpoint force and immediately retracted (Fig. 5a) For static compression, the bead was lowered as described for transient compression, but the setpoint force was applied for 60 s, after which the cantilever was retracted (Fig. 5b).

A single transient compression of the soma by 5 kPa, which can evoke single action potentials, did not change the mean firing rate of a neuron (Fig. 5c). We then applied static compression at two considerably different pressures of 0.1 kPa and 5 kPa (Supplementary Note 1). No differences in the mean firing rate of neurons during or after compression at both pressures were observed (Fig. 5d and Supplementary Fig. 9). Moreover, no changes in inter-spike intervals were observed during static compression (Supplementary Fig. 10a,b). To further assess whether rapidly changing pressures affected the neuronal activity, the neuronal soma was subjected to three successive transient compressions of 5 kPa. On average, three repeated transient compressions increased the neuronal firing rate by 25% (Fig. 5e). Furthermore, neurons indented once transiently at 0.1 µm s^–1^ until reaching 5 kPa increased their spontaneous firing rate by 12% (Fig. 5f). Importantly, no neuronal injury or cell death was observed after mechanical stimulation (Supplementary Videos 3 and 4 and Supplementary Note 2).

The results demonstrate an impressive resilience of spontaneously active neurons to considerable static mechanical compression of their soma. A single transient compression of the same magnitude did not change the spontaneous activity of neurons. However, by repetitively and transiently compressing the soma with the same pressure applied as for static compression, or at a slow indentation speed of 0.1 µm s^–1^, the neurons increased their firing rate. This finding suggests that cortical neurons differentiate and respond to distinct characteristics of mechanical stimuli such as magnitude, speed and rapidly changing pressures.

## Mechanical compression effects neuronal waveform

We characterized to what extent mechanical compression changes the neuronal morphology and the action potential waveform features. The soma of cultured rat cortical neurons showed mean heights of 8.10 µm (s.d., 1.31 µm) (Supplementary Fig. 11a,b). To visualize the morphological changes associated with mechanical compression, cortical neurons were seeded on glass coverslips, indented with the 5 µm bead, and imaged by confocal microscopy (Fig. 5g). Local indentation with 0.1 kPa or 5 kPa compressed the soma by 0.88 µm (s.d., 0.31 µm) or 5.63 µm (s.d., 0.96 µm) (Supplementary Fig. 11c,d). The neurons did not show any blebbing or membrane damage during static compression for 60 s. During 5 kPa compression, the diameter of the soma increased by 3.95 µm (s.d., 1.01 µm) (Fig. 5g–i).

The spike-sorted HD-MEA data were categorized into before, during and after compression, and mean waveform features for each period extracted (Supplementary Fig. 10c–e). Upon static compression with 0.1 kPa, the spike properties on the ‘target’ electrode located under the compression site did not change (Supplementary Fig. 10f,g). However, upon static compression with 5 kPa, the underlying target electrode recorded increasing spike amplitudes (Supplementary Fig. 10h). Such change was expected as the compression physically deforms the soma, and considerably increases the contact area and seal resistance between the neuronal membrane and underlying target electrode. Although we cannot completely rule out the effect of compression (diameter change) and physical movements of the soma during compression on the target electrode, a 1.13-fold spike amplitude increase at electrodes ∼50 µm away from the target electrode suggests that ion channels change activity during 5 kPa compression (Fig. 5j and Supplementary Fig. 10j,k). While the spike amplitude increased, the halfwidth of the waveform changed neither on the target nor on distant electrodes (Supplementary Fig. 12a–f).

It has previously been shown that a negative pressure ∼30 mm Hg (∼4 kPa) applied through a patch pipette increases the amplitude of the sodium current ∼1.5-fold, thus implying mechanosensitivity of Na_v_1.5 ion channels, which is reversible^39^. Another study corroborated the mechanosensitivity of Na_v_1.5 channels, showing that their unitary conductance and maximum opening probability were not affected by applying static negative pressures of 30–120 mm Hg (∼4–16 kPa), but that the peak current increased through activating an increasing number of channels during stress application^40^. Multiple functional isoforms of Na_v_1.5 channels are expressed in rat cortical neurons^41^. While most studies showing mechanosensitivity of Na_v_1.5 channels were conducted on HEK293-cell-attached patches, the distinct increase in spike amplitudes observed here at 5 kPa indicates that these ion channels in primary neurons change activity upon static compression.

## Conclusions

Here, we introduce a unique approach to simultaneously measure the mechanical properties, apply mechanical stimuli and monitor the electrophysiological activity of individual neurons within networks. No correlation between the stiffness of neuronal compartments, such as soma and neurites, with the spontaneous activity (firing rate) of neurons is found. Such correlations may be expected, given that neuronal activity is typically enhanced on stiffer substrates^30^. Our measurements thus suggest that changes in mechanical properties correlating with neuronal activity must be very small and probably occur at the basal side of the cell, which is inaccessible to AFM^42^. Notably, estimating the Young’s modulus from cell stiffness measurements has shortcomings^42^ and does not recapitulate the viscoelastic properties of neurons^43^. Importantly, the mechanical properties of biological molecules, cells and cellular systems depend on the frequency or speed at which they are probed^44^. Thus, a thorough investigation would require extensive rheological measurements rather than Young’s modulus estimations conducted at only one frequency or speed. The AFM–HD-MEA approach introduced here could facilitate the seamless integration of AFM-based microrheology techniques^43,45^.

Transient mechanical stimuli of the soma can evoke action potentials in cortical neurons, which propagate along neurons and neuronal networks similarly to spontaneously generated potentials. Thus, cortical neurons can convert local mechanical stimuli into electrophysiological signals. Identifying the ion channels involved in such mechanically evoked responses requires high-throughput screening^11,38^. However, the generation of mechanically evoked action potentials requires that the neuronal membrane is anchored to the underlying actin cytoskeleton. Whereas the soma of cortical neurons is functionally sensitive to mechanical stimuli, it exhibits impressive resilience to static mechanical compression. Even upon compressing the soma to 20% of its thickness, the firing rate of spontaneously active neurons does not alter. Yet, exposing the soma to repeated transient compressions of the same magnitude as applied for static compression alters the firing rate of neurons within the network. We also observe that neuronal soma respond very differently to the speed of mechanical stimuli. Whereas very slow mechanical indentations (0.1 µm s^–1^) evoke bursts of neuronal responses, faster indentations stimulate responses at lower probability. Additionally, cortical neurons show higher sensitivity to lower indentation forces (pressure) if applied at lower speed. These findings highlight that individual neurons sense the magnitude and the temporal features of mechanical stimuli.

In the present work, we focused on somas and dendrites of cultured neurons; axons are usually thinner than dendrites and buried in the cultures, and therefore cannot be probed by beaded microcantilevers. Using microfluidic channel or barrier structures and patterning extracellular matrix proteins on HD-MEA chips will probably make axons mechanically accessible. Previous reports used HD-MEAs to distinguish the velocity of action potentials in healthy and diseased states of human induced pluripotent stem cells^46^. Correlating the viscoelastic properties of axons with velocities of action potential propagation using our multiparametric method could address several interesting and long-standing hypotheses on the electromechanics of action potential generation^47^. Proof-of-principle experiments in which we measured the stiffness of a cerebellar slice while visualizing single cells and recorded the corresponding electrophysiological data (Supplementary Fig. 13) show that our method can be extended to tissue preparations. Thus, a door has been opened to explore new ways of mechanical characterization, stimulation and control of complex electrogenic biological systems in organoids and tissues.

## Methods

### Primary neuron culture preparation

The experimental protocols involving animal tissue harvesting were approved by the veterinary office of the Canton Basel-Stadt according to Swiss federal laws on animal welfare and were carried out in accordance with the approved guidelines. HD-MEA chips or glass coverslips were sterilized in 70% ethanol for 60 min and washed with sterile deionized water under laminar airflow. The electrode array or the coverslips were then treated with 10 µl of 0.05% (v/v) poly(ethyleneimine) (Sigma-Aldrich) in borate buffer (Thermo Fisher Scientific) for 40 min at room temperature and washed with deionized water, followed by incubation with 8 µl of 0.02 mg ml^–1^ laminin (Sigma-Aldrich) in neurobasal (NB) medium (Gibco) for 30 min at 37 °C. E-18 Wistar rat embryos were dissected in ice-cold HBSS (Gibco) to harvest their cortices, which were then dissociated in 0.25% trypsin–EDTA (Gibco). The dissociated cortical cells were gently triturated and filtered through a 0.45 µm sieve to obtain single cells. Cell density was estimated, and 10 µl of 3,000 cells µl^–1^ solution was plated on the electrode array. Cells were allowed to attach to the arrays by incubating the chips at 37 °C for 40 min before adding 2 ml of NB plating medium. NB plating medium was prepared by adding 50 ml horse serum (HyClone), 1.25 ml glutamax (Invitrogen) and 10 ml B-27 (Invitrogen) to 450 ml Neurobasal (Gibco). The HD-MEA chips were kept inside a cell culture incubator at 37 °C and 5% CO_2_. After 3 days, the cells were cultured in serum-free NB plating medium up to DIV 20 by exchanging 50% of culture media with fresh NB plating medium once every 3 days. All experiments, except for the data shown in Fig. 1i, were conducted between DIV 14 and 16 because cells showed different mechanical properties while growing^11^. The experiments for the data shown in Fig. 1i were collected between DIV 22 and 24, when the neuronal networks showed synchronous bursting activity.

### Cerebellum slice preparation

Wild-type mice (postnatal day 14 C57BL/6Rj, Janvier Labs) were decapitated under isoflurane anaesthesia; their brains were removed and immersed into ice-cold carbogen-bubbled (95% O_2_ + 5% CO_2_) artificial cerebrospinal fluid (aCSF) solution. Sagittal cerebellar slices of ∼350 µm were obtained using a vibratome (VT1200S, Leica). All slices were maintained at room temperature in aCSF until use. The aCSF was composed of 125 mM NaCl, 2.5 mM KCl, 2 mM CaCl_2_, 1 mM MgCl_2_, 25 mM glucose, 1.25 mM NaH_2_PO_4_ and 25 mM NaHCO_3_. The electrode array of the HD-MEA chips was then treated with 10 µl of 0.05% (v/v) poly(ethyleneimine) (Sigma-Aldrich) in borate buffer (Thermo Fisher Scientific) for 40 min at room temperature and washed with deionized water, followed by an incubation with 8 µl of 0.02 mg ml^–1^ laminin (Sigma-Aldrich) in Neurobasal medium (Gibco) for 30 min at 37 °C. Excess laminin after incubation was pipetted out, and the array was allowed to dry. The cerebellar slice was then gently placed on the electrode array using a cut pipette tip to avoid damage caused by shear forces during pipetting (Supplementary Fig. 13). A 2 mm custom-made harp was placed on the tissue slice to immobilize the tissue slice. Tissue slices were then immersed in aCSF by gently adding drop by drop. The immobilized tissue slice immersed in aCSF was then allowed to adhere to the electrode array for 30 min. Just before the recording, the harp was removed, and the AFM head was mounted onto the HD-MEA chip. The tissue was continuously perfused with carbogen-bubbled aCSF to maintain cell viability and activity.

### HD-MEA set-up

Complementary-metal-oxide-semiconductor (CMOS)-based HD-MEA chips featuring 26,400 electrodes (17.5 µm pitch) within an overall sensing area of 3.85 × 2.10 mm^2^ were used^25^. The chips were fabricated in a commercial foundry and post-processed and packaged in house (Supplementary Fig. 1b). Transparent polycarbonate rings (GB Plex) of 4 cm diameter were glued onto the chips, and the wire bonds were encapsulated with biocompatible dark epoxy (EPO-TEK 353 ND). The platinum-black coating of the electrodes was electrodeposited to decrease the electrode impedance and improve the signal-to-noise characteristics (Supplementary Fig. 1c). Commercial versions of our custom-developed HD-MEA system can be purchased (MaxOne model) from MaxWell Biosystems.

### Sample holder and stage heater

A thermoelectric Peltier-based sample heater, with a temperature probe for closed-loop feedback, was controlled by a home-built temperature controller to maintain the sample at 37 °C. The sample heater was aligned with the thermal conduction pad on the HD-MEA chip. The chips were then mounted onto a sample holder and locked in position with the holder’s spring-loaded pin. Prusa I3 MK3S+ was used to 3D print the frugal syringe holders for the perfusion set-up which were attached to the sample holder. The entire set-up was mounted onto a custom-made *x*,*y* piezo stage via a base plate.

### *x*,*y* piezo stage

Two linear piezo stages (Xeryon XLS-1 series) with an encoder resolution of 5 nm were attached to each other perpendicularly (Supplementary Fig. 1a). The linear stages were controlled by a Xeryon XD-M multiaxis controller connected to a LabVIEW-based user interface. The top left electrode of the HD-MEA chip was registered as the origin of the coordinate system, and the electrode coordinates with the neurons of interest were extracted from the MaxWell Biosystems user interface. These coordinates were then fed to the XD-M controller using custom scripts for easy positioning of neurons under the AFM cantilever.

### Long-working-distance fluorescence microscopy

An optical microscope (Nikon SMZ 25) with 15.75× adjustable zooming lenses and 1× objective (Nikon, MNH55100 P2-SHR PlanApo 1X; numerical aperture, 0.156) was aligned with the set-up as mentioned in main text. A TTL-controllable light-emitting diode (LED) illuminator (CoolLED PE 300^ultra^) was used as a light source for excitation/emission filter-free imaging. A triple bandpass beam splitter (F66-412, AHF Analysentechnik) was used to filter the reflected excitation light from the HD-MEA chip. A large CMOS array camera (Nikon DS-QI2) with 4,908 × 3,264 pixels (pixel size, 7.3 × 7.3 µm^2^) was mounted onto the microscope using a 2.5× f-mounted projector lens allowing for sampling of up to 45 f.p.s. with a final magnification of ∼40× and a resolution of 0.46 µm per pixel.

### AFM

An AFM head (Catalyst, Bruker) was mounted and aligned with the set-up as mentioned in the main text. A 15 µm piezo scanner on the head was used to collect all force–displacement and force–time curves, while a 150 µm piezo was used to position the cantilever on the neuron. The data were collected and exported to .txt files using the AFM software (Nanoscope v.9.2, Bruker). The data were analysed and plotted with Python scripts. Silica beads with 5 μm diameter (Kisker Biotech) were glued to the free end of tipless microcantilevers (CSC-37 or 38, Micromash HQ) using ultraviolet glue (Dymax) and were ultraviolet cured for 20 min. Beaded cantilevers were cleaned for 5 min using a plasma cleaner (Harrick Plasma), then mounted onto the fluid probe holder with a 2 mm sapphire window, and calibrated using the thermal noise method^48^.

### Correlative AFM, HD-MEA and optical microscopy

The AFM, HD-MEA and optical microscope were aligned as shown (Fig. 1a). Fluorescence light from neurons on HD-MEA chips was collected through a sapphire window in the AFM cantilever holder and passed to the optical microscope. The fluorescence image of the neurons on the HD-MEA chip were sequentially collected in regions of interest, stitched and registered on the Maxwell MEA user interface to localize neurons on the HD-MEA chip (Supplementary Fig. 2a–d). The *x*,*y* coordinates of neurons of interest were then fed to the *x*,*y* stage via LabVIEW scripts, thus placing the AFM cantilever at the desired positions with ∼5 nm precision. The entire set-up was installed on a damping isolated table and placed in a noise-protected, temperature-controlled chamber to reduce mechanical noise and thermal drift.

### TTL synchronization

TTL pulses from DS-Qi2 were extracted using a home-built connector with a 3.5 inch four-pole pin, a mini plug on one side and a female 24 AWG jumper (RND 255-00015, Distrelec) on the other side. Pin 1 was the ground and pin 4 (EXP_TMG) received 2.4 V on HI (high) level at live operation according to the set exposure time. A negative pulse of 0–5 V was extracted from the front panel output channel 1 of the AFM controller (Bruker Nanoscope V) through a home-built connector with a standard BNC male pin on one side and a female 24 AWG jumper (RND 255-00015, Distrelec) on the other side. The signals, extracted from both camera and AFM, were then routed to pin 2 and 8 of a single high-speed optocoupler. The signals were then sent to the HD-MEA data-acquisition system via a field-programmable gate array, providing precise time stamps of the events detected by AFM and optical microscopy on raw file recordings of HD-MEA data collected at 20 kHz.

### Stiffness tracking protocol and measurements

The apparent Young’s modulus was calculated from an average of five force–displacement curves collected on the neuronal soma. We then waited for 30 s to ensure that there were no measurement-induced mechanical changes in the neurons and collected five force–displacement curves again (Fig. 2a), which we labelled as one measurement cycle and which is represented by one dot (Fig. 2b). We repeated this measurement cycle up to 5 min on a neuron and moved to the next neuron in the network. Sixty minutes after the first measurement on the first neuron, we returned to the same neuron, and the measurement cycle was repeated. The measurements comprised a long-time-scale-tracking cycle. This long-time-scale tracking cycle was repeated three times (Fig. 2c). For stiffness measurements of neurites, we first recorded the electrophysiological activity of the neurons for 5 min and then identified two well-isolated neurites per neuron and collected force–displacement curves on them. Force–displacement curves were collected using CSC-38 microcantilevers (nominal spring constant, ∼0.02 N m^–1^) featuring 5-μm-diameter beads as mentioned above. A maximal force of 700 pN on somas and of 400 pN on neurites was used to collect force–distance curves (Supplementary Fig. 14a,b). For all the measurements, the tip velocity was kept constant at 10 µm s^–1^. The contact point was determined from the approach force–displacement curve as the *x* intercept at a value of force, which was five times higher than the standard deviation of baseline noise, followed by a manual curation. Indentation depth was calculated as the displacement value at the contact point after subtracting the cantilever deflection and setting the displacement value at the maximal force to zero in the force–displacement curve (Supplementary Fig. 14c). The indentation depth for the same given maximal force would vary from soma to soma depending on its stiffness. Therefore, we have set an indentation depth cut-off of 750 nm. In the force–displacement curves, all force values for which the corresponding displacement value exceeded the indentation depth cut-off were discarded. The apparent Young’s modulus was calculated from such curves to which the Hertz model for a spherical indenter with an elastic half-space^49^ was fitted using custom codes in Python.

The Young’s modulus values measured in our study for the soma of neurons are in a comparable range with previous reports^10,11^. However, the Young’s modulus of the neurites is lower than the reported values, while they still are on a comparable order of magnitude. This bias might result from our choice to measure the two thickest basal neurites of the neuron, which are typically softer. For measuring the stiffness of the neuronal somas during burst and IBIs, we have collected force–displacement curves at a frequency of 5 Hz. This process was repeated several times for each soma, and the average stiffness of the soma during bursting periods and during IBIs was calculated. While the bursts usually appear in a rhythmic pattern (Fig. 2h), it is difficult to predict for cultured neurons when a single neuron undergoes bursting. To collect at least two force–displacement curves during bursting periods or IBIs, while minimizing the number of times the probe comes in contact with the neuron, we indented the neuron 5 times s^−1^.

### Static compression protocol

A 5-µm-diameter bead, glued to a tipless AFM microcantilever (CSC-37; nominal spring constant, ∼0.8 N m^–1^), was positioned above the soma. The bead was lowered onto the soma until a setpoint force required to apply 0.1 kPa or 5 kPa was reached and kept in contact for 60 s using constant-height feedback before retracting the bead (Fig. 5b). The force–time curve recorded at 500 kHz sampling rate shows both the vertical displacement of the AFM head and the force response of the soma. The average height of soma of rat cortical neurons was ∼8 µm (Supplementary Fig. 11a).

### Functional calcium imaging

Genetically encoded calcium sensors were expressed in neurons using adeno-associated viruses (AAVs). AAV1-EF1a-GCaMP6s (1.8 × 10^13^ viral genomes ml^−1^) were used at a multiplicity of infections of 5.0 × 10^4^ to express GCaMP6s. Neurons were infected at DIV 3; expression was usually seen at DIV 5–9.

### Functional calcium imaging analysis of mechanically stimulated neurons

An average fluorescence intensity curve Δ*I*/*I* of the GCaMP6s curve was calculated as the mean signal over the entire image relative to the baseline of the image. Peak detection in the signal was performed by finding local maxima within a 3 s window. An event was marked as the start of a neuronal response when the calcium signal amplitude reached 10% of the peak value. Data from 2.5 s before and 10 s after the start of the response peak (*t* = 0) was plotted.

### HD-MEA recordings

The MaxWell Biosystem user interface (MaxLab Live v.22.13) was used to record the data. The whole-chip fluorescence image was registered on the user interface (Supplementary Fig. 2c). Neurons of interest were identified from calcium spikes, and spiking activity (action potentials) was obtained from the live raster plots on the user interface. Once a neuron of interest was identified, 512 electrodes around this neuron in a rectangular configuration were routed to the readout. After positioning the AFM cantilever on the neuron, the channels were offset five times to compensate the noise from the infrared laser used by the AFM to detect the cantilever deflection before starting the recording. A gain of 512 and a high-pass filter with a cut-off at 300 Hz were used for all recordings. To improve the performance of the spike-sorting algorithms in Fig. 5, neuronal action potentials were recorded 2.5 min before and after mechanical stimulation.

### HD-MEA data analysis

All collected HD-MEA data were processed via custom scripts based on Spikeinterface^50^. Briefly, extracellular recordings were filtered and spike-sorted using Kilosort2, followed by manual curation of all recordings. A conservative inter-spike interval violation threshold of 0.5 and a signal-to-noise ratio threshold of 5.0 were used for curation. Template similarity, auto-correlograms and cross-correlograms were used for unit quality assessment. Waveform features such as halfwidth and repolarization slope were extracted for spike-sorted units for the respective epochs with Python scripts using functions from Spikeinterface (Supplementary Fig. 12a–i). The relative changes in the waveform features on all electrodes within the extracellular footprint of a neuron were obtained by dividing the value of the mean waveform feature of all spikes during mechanical compression by the mean waveform feature of all spikes before compression. Mean firing rate and inter-spike interval were computed from the extracted spike trains using the Elephant electrophysiology analysis toolkit 0.11.2^51^. The mean firing rates for stiffness correlation data were computed by placing the spikes in 30 s bins to match with the time points of the stiffness values. The mean firing rates for the compression protocol were computed by placing spikes in three bins of before, during and after compression.

### Combined AFM and confocal microscopy

Time-lapse confocal imaging was performed on an inverted laser-scanning confocal microscope (Observer Z1, LSM 700; Zeiss) equipped with a 25×/0.8 LCI PlanApo water immersion objective (Zeiss). An AFM (CellHesion 200; JPK Instruments) was mounted onto the confocal microscope. Mechanical compression protocols were executed using JPK CellHesion software. For mechanical stimulation, AFM was used to approach the cantilever with the bead onto the cell at speeds of 0.1, 1, 10 or 100 μm s^–1^ until reaching the setpoint force, and, thereafter, immediately retracted at the same speed as used for the approach. For experiments determining the threshold force to mechanically stimulate neurons, the applied setpoint force was stepwise increased from 50 to 400 nN in 50 nN increments and intervals of 20 s (Supplementary Fig. 6). The setpoint force of the approaching bead was stepwise increased until a neuronal response was recorded. After successful stimulation of the neuron, the cantilever was retracted, and a new neuron was selected for stimulation.

### Statistical analysis

All data showing waveform properties were tested for normality with the Shapiro–Wilk test and *Q*–*Q* plots. All data groups were not normally distributed and were dependent data groups. Therefore, we used the Wilcoxon signed-rank test. The null hypothesis was that there is no difference in medians between the pairwise compared distributions. *P* values >0.05 were considered non-significant. The waveform features were extracted from the averaged waveform of each neuron obtained from *n* > 5,000 spikes. All data showing mean firing rates were compared with the Wilcoxon signed-rank test from *n* > 10,000 spikes. AFM data groups were compared using the two-tailed Mann–Whitney *U*-test. *P* values for each comparison are mentioned in the figure legends. Pearson correlation was used to determine the linear correlations in stiffness and mean firing rate values. No statistical methods were used to predetermine sample sizes. Data collection and analysis were not performed blind to the conditions of the experiments.

### Reporting summary

Further information on research design is available in the Nature Portfolio Reporting Summary linked to this article.

## Online content

Any methods, additional references, Nature Portfolio reporting summaries, source data, extended data, supplementary information, acknowledgements, peer review information; details of author contributions and competing interests; and statements of data and code availability are available at 10.1038/s41565-024-01609-1.

### Supplementary information

Supplementary Information Supplementary Figs. 1–14 and Note 1.

Reporting Summary

Supplementary Video 1 Time-lapse imaging of spontaneously firing neuronal networks expressing GCaMP6s on HD-MEA chips.

Supplementary Video 2 Time-lapse confocal imaging of neuron subjected to transient compression of 5 kPa on the soma.

Supplementary Video 3 Time-lapse confocal imaging of neuron subjected to a static compression of 5 kPa on the soma.

Supplementary Video 4 Time-lapse confocal imaging of neuron subjected to a static compression of 0.1 kPa on the soma.

### Source data

Source Data Figs. 2–5 Statistical source data for Figs. 2–5.

## Data Availability

The relevant raw data for this study are available for research purposes from the corresponding authors upon reasonable request. Source data are provided with this paper.

## Code availability

All custom code used to analyse the data in this study is available for research purposes from the corresponding authors upon reasonable request.
