# Supplementary material for: Mechanical stimulation and electrophysiological monitoring at subcellular resolution reveals differential mechanosensation of neurons within networks
Source: Nat Nanotechnol. 2024 Feb 20;19(6):825–33. doi: 10.1038/s41565-024-01609-1 (PMC11186759; doi:10.1038/s41565-024-01609-1)
Supplement: Supplementary file 1 — Supplementary Figs. 1–14 and Note 1. [file 41565_2024_1609_MOESM1_ESM.pdf]

nature nanotechnology

Article

https://doi.org/10.1038/s41565-024-01609-1

Mechanical stimulation and electrophysiological monitoring at subcellular resolution reveals differential mechanosensation of neurons within networks

In the format provided by the authors and unedited

SUPPLEMENTARY FIGURES

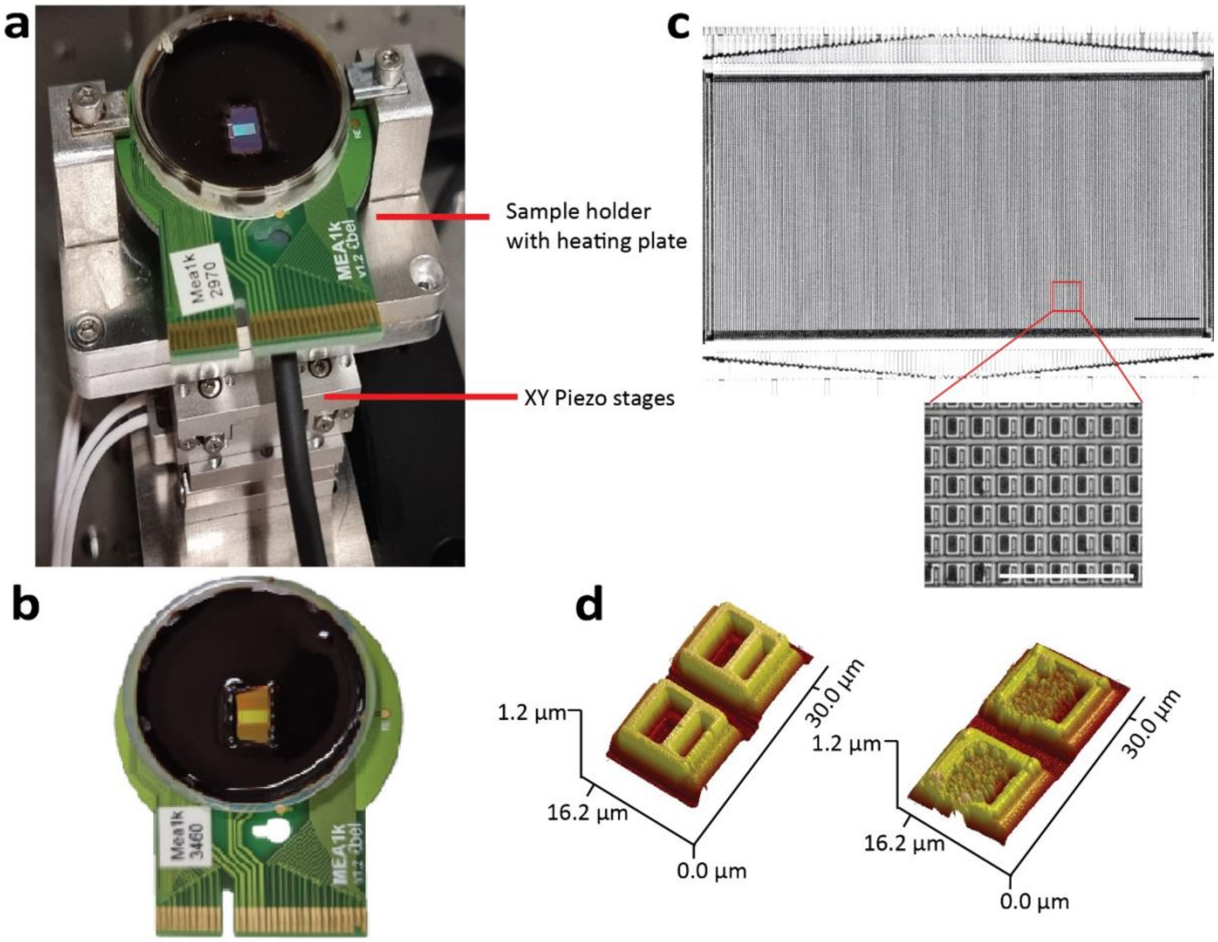

Supplementary Figure 1. **Adaptation of the HD-MEA chip to the AFM and light microscopy setup. a**, Sample holder and XY piezo stage holding the HD-MEA chip. **b,** HD-MEA chip encapsulated with dark (black) epoxy. **c,** Brightfield image of the electrode array of the HD-MEA chip. Scale bar, 500 µm. The zoom in of the region highlighted by the red box shows single HD-MEA electrodes. Scale bar, 105 µm. **d,** AFM topography of two HD-MEA electrodes before and after Pt-black deposition.

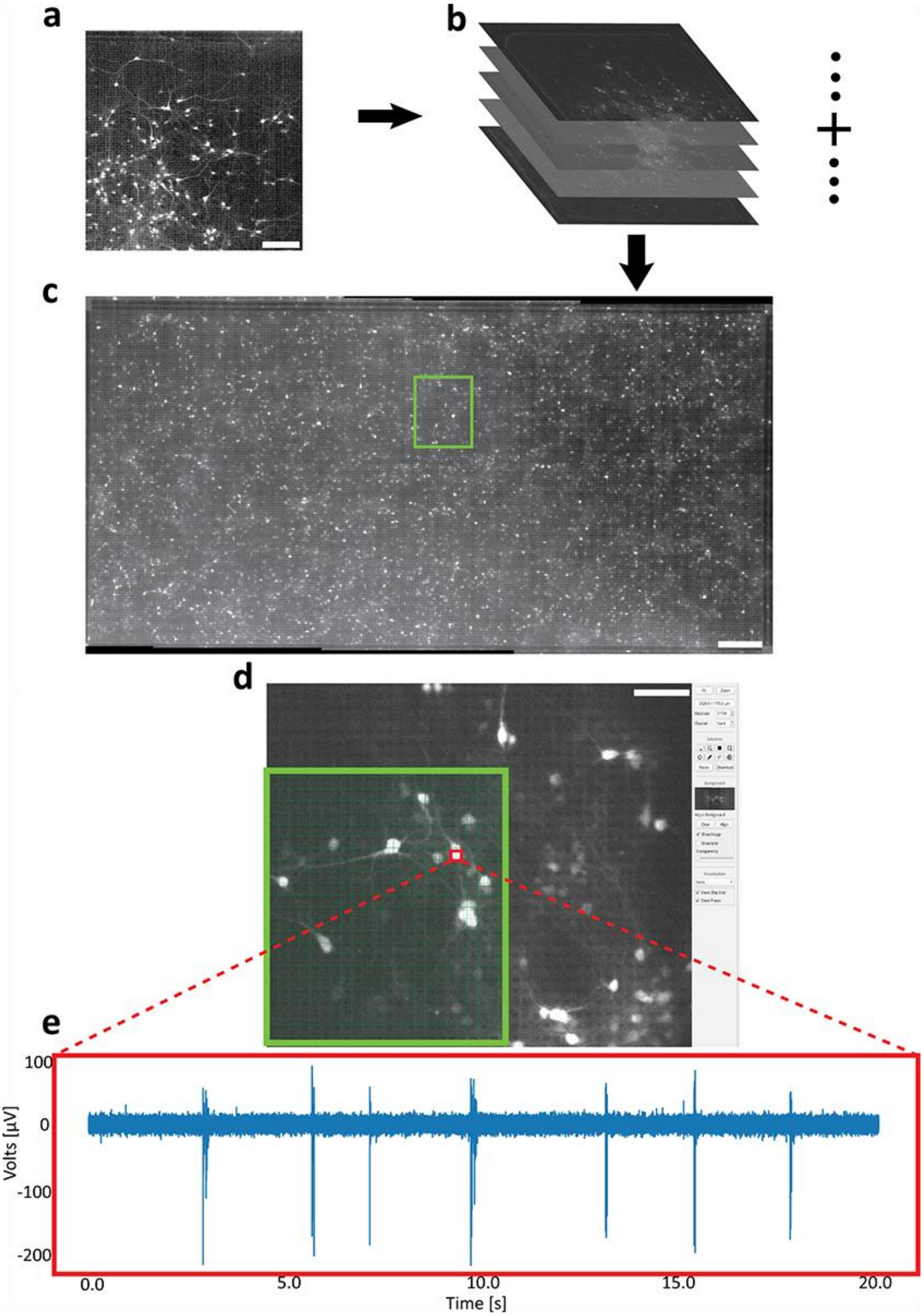

Supplementary Figure 2. **Set-up of the HD-MEA recordings. a**, Fluorescence image of neurons, which membranes were stained with NeuO live cell stain. **b,** A representation showing stitching of all fluorescence images taken in sequence for the image in **a**. **c,** Stitched fluorescence image of the entire HD-MEA electrode array with rat cortical neurons. Scale bar, 300 µm. **d,** Zoom-in of the image in **c**, after registration on the HD-MEA user interface. **e**, Filtered, raw voltage trace depicting neuronal spikes of the electrode highlighted in **d,** shows the high signal-to-noise ratio.

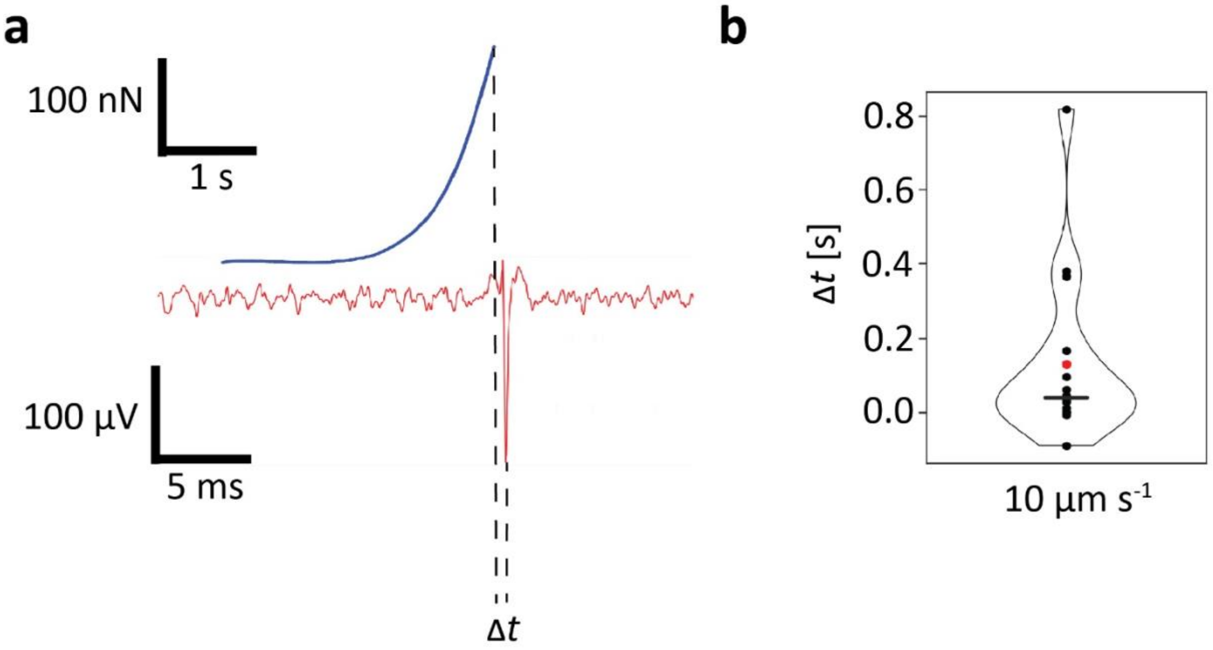

Supplementary Figure 3. Time delay between reaching the setpoint of the mechanical stimulation force and spike time of the neuronal response. a, Time dependence of the mechanical stimulation force applied on the soma of a cortical neuron (blue) and its extracellular potential (red). The neurons were indented with a 5 µm diameter silica bead glued to the AFM cantilever by applying a force of 200 nN (≈ 5 kPa) at an indentation speed of 10 m s^{-1}. Δ*t* gives the time difference (two dashed lines) between the time point, when the setpoint force was reached and the time point at which the peak of the extracellular potential was detected. **b**, Δ*t* for mechanical indentations of 200 nN (≈ 5 kPa) at 10 µm s^{-1}. Black dots represent the data of n = 15 individual stimulations from $n_{neurons}$ = 8 independent neurons, red dot the mean, and black line the median.

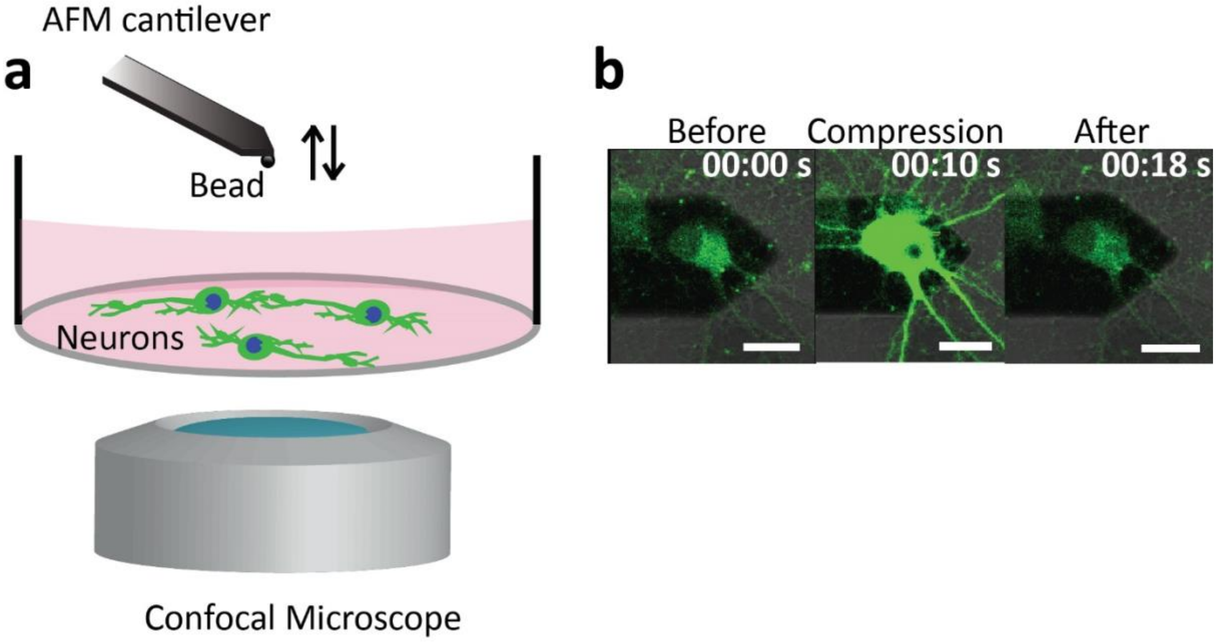

Supplementary Figure 4. Mechanical stimulation of cortical neurons using an AFM combined with confocal microscopy. a, A 5 µm diameter bead glued to the free end of the AFM cantilever is used for transient mechanical indention of soma of a cortical neuron at 10 µm s^{-1} (**Methods**). The neuronal response to this mechanical stimulation is read out through functional calcium imaging by confocal microscopy. **b,** Overlaid DIC and fluorescence images of the AFM cantilever (shadow) and a cortical neuron expressing GCaMP6s (green) before, during, and after 5 kPa mechanical stimulation. To isolate mechanically evoked responses of the neurons, their spontaneous activity was blocked with glutamate receptor antagonists 10 µM DNQX and 40 µM D-AP5. Scale bars, 20 µm.

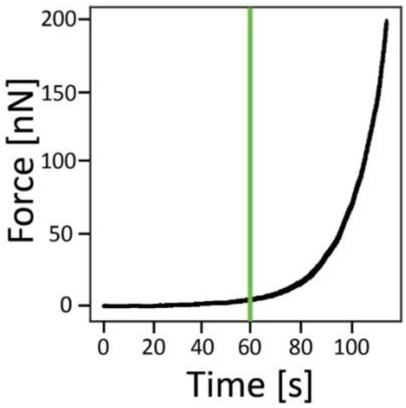

Supplementary Figure 5. Force-time curve obtained from indenting the soma of a cortical neuron at a speed of 0.1 µm s^{-1}. Black line shows averaged force over time curve recorded upon indenting a neuron ($n_{neurons}$ = 10) with a 5 µm diameter bead at a speed of 0.1 µm s^{-1} until reaching a setpoint force of 200 nN. The vertical green line indicates the contact point of the bead and neuron. Time under compression is computed as the time taken from the contact point (≈60 s, green line) to reach the setpoint force of 200 nN (≈120 s).

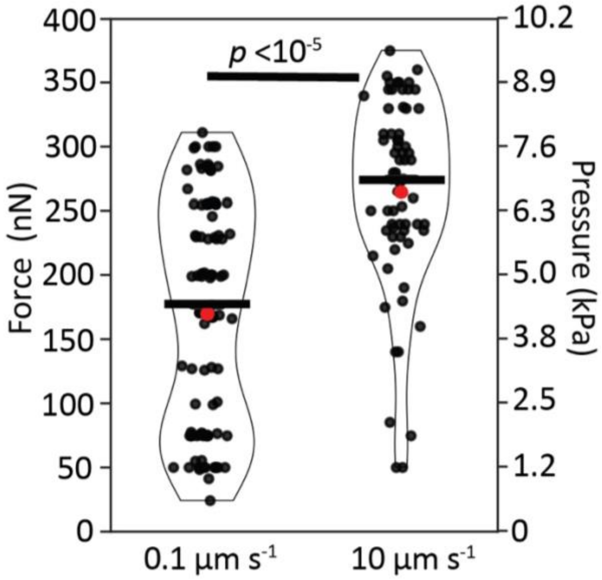

Supplementary Figure 6. Threshold forces / pressures required to mechanically evoke neuronal responses depend on the speed at which the bead is indenting the neuronal soma. Threshold forces / pressures at which cortical neurons respond to the mechanical stimulation depends on the indentation speed of 0.1 µm s^{-1} ($n_{neurons}$ = 85) and 10 µm s^{-1} ($n_{neurons}$ = 68). In the experiment, the individual neurons were mechanically indented through stepwise increasing forces from 10 to 400 nN. Black dots represent single neurons, red dots the mean, and black lines the median. Two-tailed Mann-Whitney test was used to compare the populations, *p*-value < 10^{-5}.

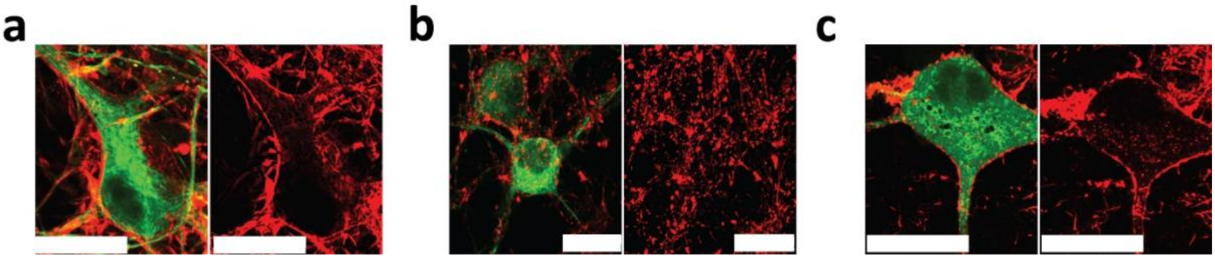

Supplementary Figure 7. Actin cytoskeleton of cortical neurons in the absence and presence of chemical perturbations. Fluorescence images of rat cortical neurons stained with SiR-Actin (red) and neuronal membrane dye NeuO (green). Left panel in each image shows a merge of red and green channels and right panel shows only the red channel. **a,** 0.1% v/v DMSO (vehicle control). **b,** Neurons incubated for more than 30 min with 0.1 µM latrunculin A. **c,** Neurons incubated for more than 30 min with 50 µM NSC668394C. Scale bars, 20 µm.

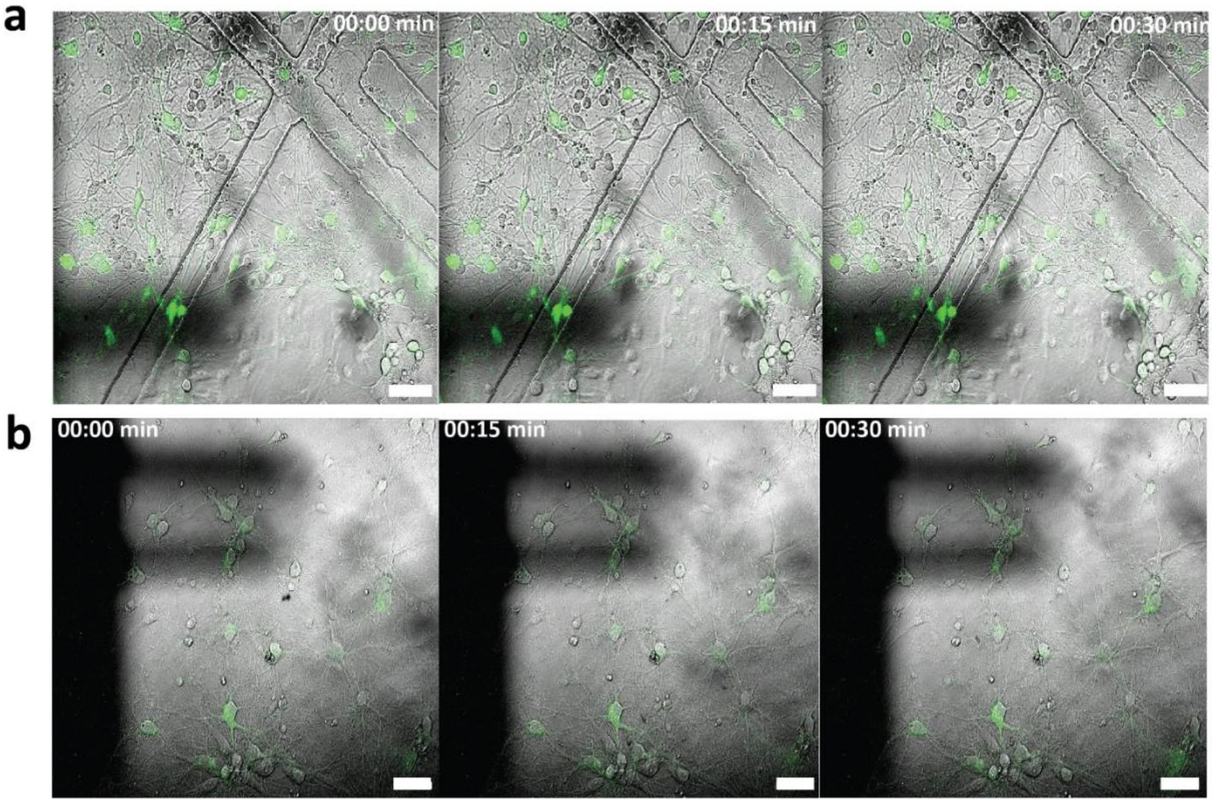

Supplementary Figure 8. Cortical neurons survive chemical perturbations. Time-lapse confocal microscopy images of cortical neurons expressing genetically encoded calcium sensor GCaMP6s and pre-treated for 60 min with **a,** 0.1 µM latrunculin A or **b,** 50 µM NSC668394C. The images show that the cortical neurons do not display any significant morphological changes (*i.e.,* blebbing). Scale bars, 50 µm**.**

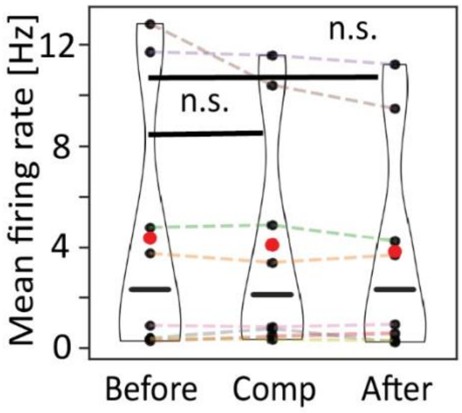

Supplementary Figure 9. Effects of static mechanical compression at 0.1 kPa on spontaneous activity of neurons. Mean firing rate of neurons before, during, and after a 60 s long static compression with 0.1 kPa ($n_{neurons} = 8$, $n_{spikes} > 1'000$). Black dots represent data from individual neurons, Red dots represent mean values and black lines represent median values. Wilcoxon signed-rank test was used to compare the data groups. n.s. represents non-significance with p-values > 0.05.

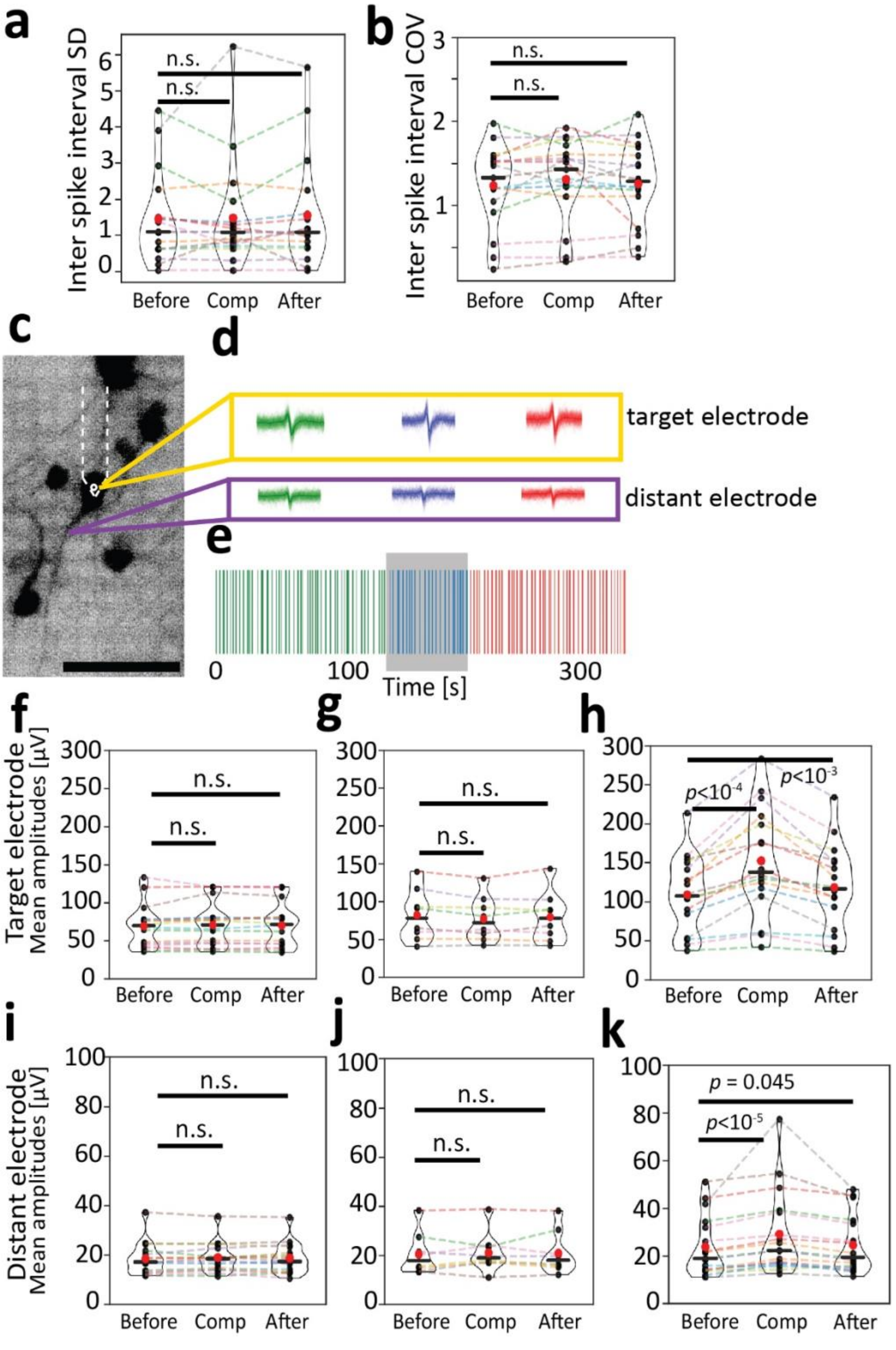

Supplementary Figure 10. **Electrophysiological responses of cortical neurons to the mechanical compression of their soma. a,b** Standard deviation and covariance of inter-spike intervals of neurons before, during and after compression with 5 kPa. **c,** Fluorescence image of rat cortical neurons on the HD-MEA chip. The neuronal membrane was stained with NeuO. White dashed lines indicate the AFM cantilever, and the white circle highlights the 5 µm diameter bead. Scale bar, 105 µm. **d**, Extracellular waveforms of the neuron in **c**, before mechanical compression (green), during compression (blue), and after compression (red). The yellow box highlights the waveforms obtained from the target electrode, where the soma was compressed, while the purple box highlights waveforms from a distant electrode. **e,** Spike times of the neuron before (green), during (blue), and after (red) compression in **c**. **f-k**,

Neuronal spike amplitudes measured on target electrode and distant electrode, before, during, and after applying different compression pressures. **f** and **i** represent control, **g** and **j** 0.1 kPa, **h** and **k** 5 kPa, with $n_{control} = 15$, $n_{0.1\ kPa} = 8$, $n_{5\ kPa} = 17$ independent neurons characterized**.** Colored dashed lines connect data points of the same neuron. The control group represents uncompressed neurons simultaneously recorded with compressed neurons. Black bars represent median and red dots mean values. Wilcoxon signed-rank test is used to compare the data groups. *p*-values are given in the figure. n.s. represents non-significance with p-values > 0.05.

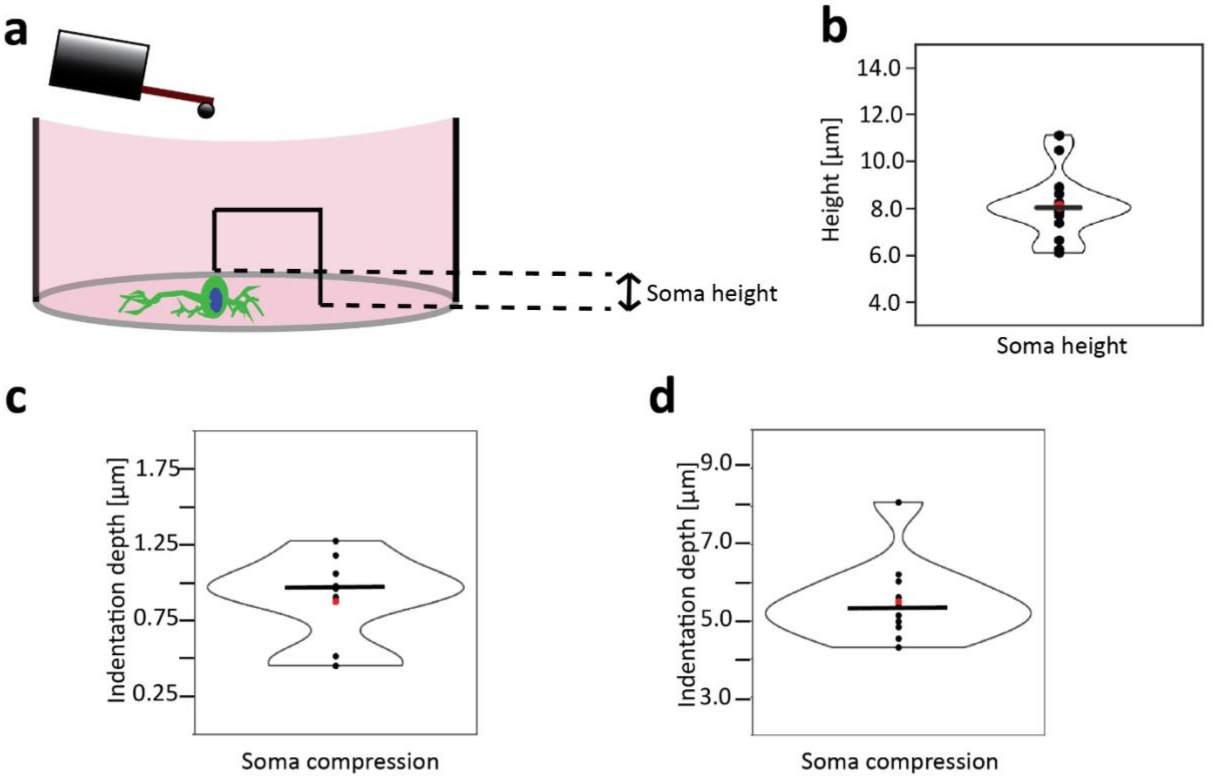

Supplementary Figure 11. Measuring the height and indentation depth of a neuronal soma by AFM. **a,** Cartoon depiction of determining the height of single cortical neurons by AFM. The solid black line shows the movement of the AFM cantilever. We measured the height of the neuron by approaching the cantilever to the soma and to the surface next to the neuron with a maximal force of ≈500 pN. **b,** Cell height measured from $n = 15$ independent cortical neurons. Each measurement was done in duplicates, and the average value of the measurements is plotted (black dots). The horizontal black line represents the median and the red dot the mean value. All height measurements were carried out on neurons seeded on glass coverslips and in a controlled environment (**Methods**). **c,d,** Indentation depth of the soma of the cortical neuron compressed with 0.1 kPa (**c**) and 5 kPa (**d**). The indentation depth was calculated from force-displacement curves collected during compressing the soma of $n = 11$ independent cortical neurons for each pressure.

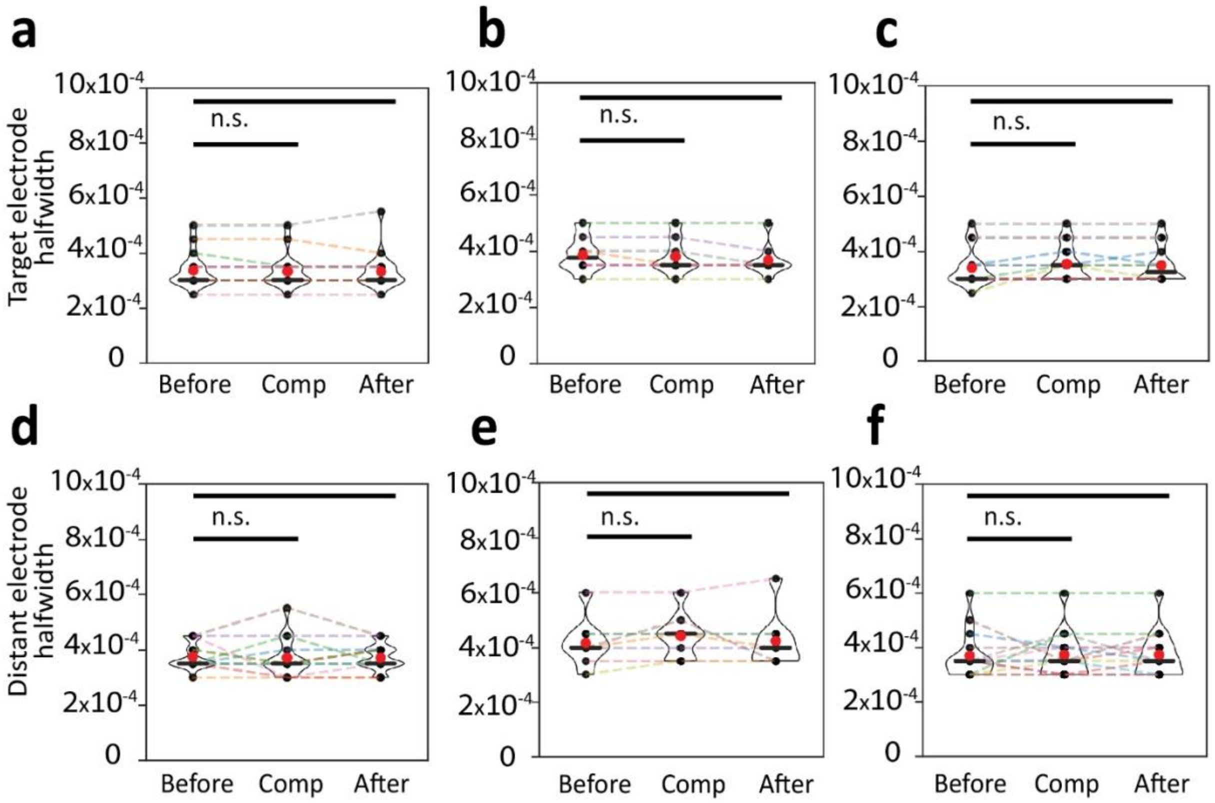

Supplementary Figure 12. Halfwidth and repolarization slopes of spike waveforms of neurons on target and distant electrodes. Halfwidth of the waveform for before, during and after compression for control, 0.1 kPa, and 5 kPa compression of the neuronal soma on the target electrode (**a, b,** and **c**) and on the distant electrode (**d, e,** and **f**). n_{control} = 15, $n_{0.1\ \text{kPa}}$ = 8, and $n_{5\ \text{kPa}}$ = 17 independently characterized cortical neurons. The control group represents mechanically unperturbed neurons simultaneously recorded with the compressed neurons. The data groups were compared using the Wilcoxon signed-rank test. n.s represents non-significant *p*-values of > 0.05. Black bars and red dots indicate median and mean values, respectively.

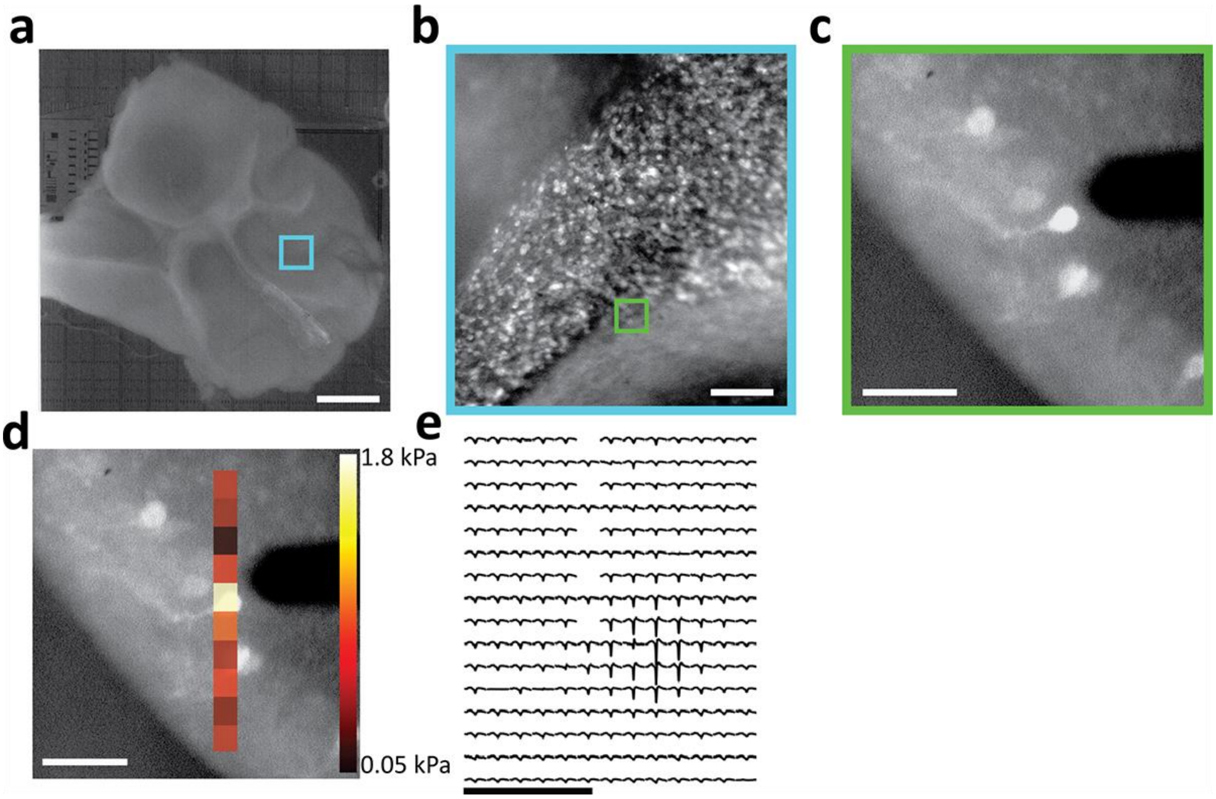

Supplementary Figure 13. Stiffness measurement and electrophysiological recording in acute mouse cerebellar slices. a, Fluorescence microscopy image of an acute mouse cerebellar slice on the HD-MEA chip. Scale bar, 1 mm. **b,** Fluorescence image of neurons stained with NeuO in the cerebellar slice highlighted by the blue box in **a**. Scale bar, 500 µm. **c,** Single neurons in the cerebellar slice highlighted by the green box in **b**, with the AFM cantilever hovering 50 µm above the sample (dark shadow). Scale bar, 50 µm. **d,** Overlayed stiffness map extracted in the region of the tissue slice shown in **c**. The Young's modulus was approximated from force-displacement curves, which had been recorded by AFM. Scale bar, 50 µm. **e,** Spike-sorted extracellular footprint of a typical cerebellar neuron at the mechanical measurement site. Scale bar, 105 µm. All measurements were carried out in a controlled environment to preserve tissue viability (**Methods**).

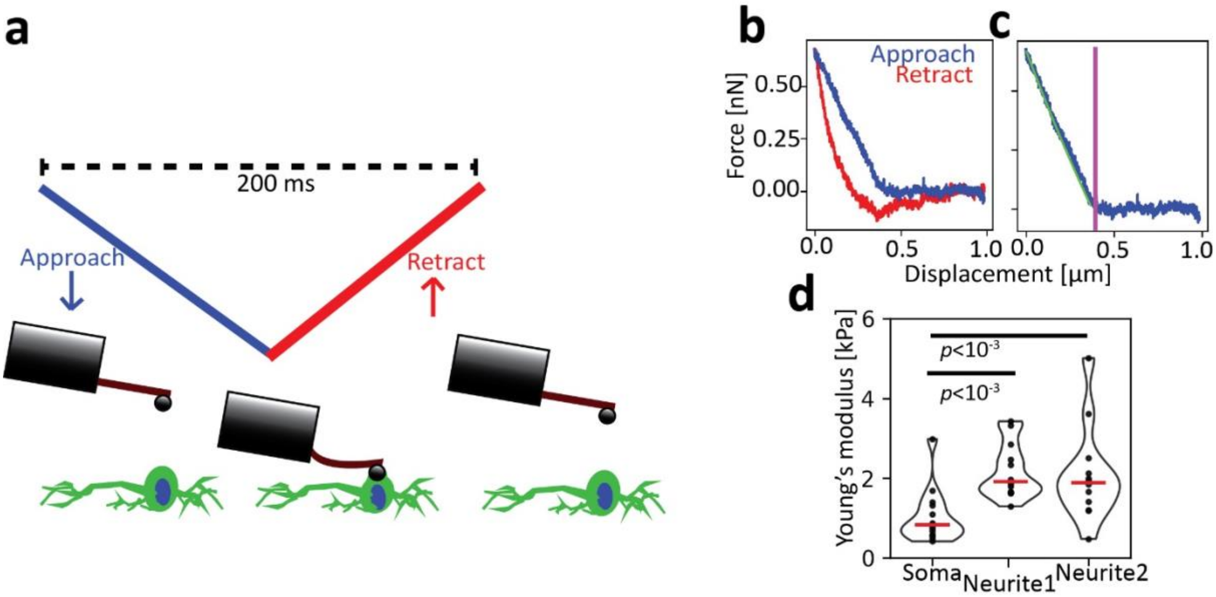

Supplementary Figure 14. **Schematic explanation of the protocol to measure Young's modulus and the compression of neurons**. **a,** A schematic of the protocol used to measure Young's modulus of neurons, showing the approach (blue line) and retraction (red line) of AFM cantilever and neuron. **b,** Typical force-displacement curves collected on a neuron. The force-displacement curve was corrected for cantilever deflection. Blue and red traces represent the approach and retraction of the cantilever bead as illustrated in **a**. **c,** The Hertz model was fitted (green line) to the approach force-displacement curve. The vertical line (magenta) indicates the contact point between bead and neuron. **d,** Apparent Young's modulus extracted from force-displacement curves collected on soma from $n = 14$ independent neurons and on neurites (neurite1 and neurite2) of $n = 12$ independent neurons. In agreement with the literature, neurites were stiffer than the soma[1,2]. Black dots represent individual averages of three force-displacement curves, and red lines indicate the median. Two-tailed Mann-Whitney test was used to compare the populations. *p*-values are given in the figure.

Supplementary Video 1. Time-lapse imaging of spontaneously firing neuronal networks expressing GCaMP6s on HD-MEA chips. Cortical neurons from E-18 Wistar rat embryos were seeded on HD-MEA chips and infected with AAVs to express GCaMP6s (**Methods**). Simultaneous functional calcium imaging and HD-MEA recordings were performed on days *in vitro* (DIV) 18. The neuronal network displays spontaneous activity. Calcium spikes and action potentials from HD-MEA recordings for an exemplary neuron of such a recording are plotted in **Fig. 1e**.

Supplementary Video 2. Time-lapse confocal imaging of neuron subjected to transient compression of 5 kPa on the soma. Time-lapse confocal fluorescence images of cortical neuron expressing GCaMP6s, mechanically compressed with 5 kPa, at an indentation speed of 10 µm s^{-1}. The neuron shows baseline fluorescence, calcium response to mechanical indentation, and return to baseline as shown in **Supplementary Fig. 4**.

Supplementary Video 3. Time-lapse confocal imaging of neuron subjected to a static compression of 5 kPa on the soma. Time-lapse confocal fluorescence images of cortical neurons expressing GCaMP6s, mechanically compressed with 5 kPa, show no active blebbing during compression or any signs of injury post compression.

Supplementary Video 4. Time-lapse confocal imaging of neuron subjected to a static compression of 0.1 kPa on the soma. Time-lapse confocal fluorescence images of cortical neurons expressing GCaMP6s, mechanically compressed with 0.1 kPa, show no active blebbing during compression or any signs of injury post compression.

Supplementary Note 1. Conversion of force values to pressure values.

In order to convert the force-threshold values to pressure values, we calculated the pressure resulting from pushing a 5 μm diameter bead onto the soma at given forces.

For compression with 200 nN:

The contact area *A* between the bead and soma is given as the half sphere surface of the bead:

$$A = 2\pi\, r^2 \qquad \text{Equation S1}$$

with *r* = 2.5 μm, the area *A* is:

$$A = 2\pi(2.5\ \mathrm{\mu m})^2 = 39.3\ \mu m^2$$

The pressure *P* is given as:

$$P = \frac{F}{A} \qquad \text{Equation S2}$$

with *F* being the indentation force. The pressure *P* corresponding to the indentation force in the discussion section were calculated in the following way. For 100 nN, *P* is:

$$P = \frac{100 * 10^{-9}\ \mathrm{kg\ m\ s^{-2}}}{39.3 * 10^{-12}\ m^2} = 2.55 * 10^3 \mathrm{kg\ m^{-1}\ s^{-2}} = 2.55\ kPa$$

Using this formula, we can obtain the pressure value of ≈ 5 kPa for 200 nN of compression force.

For compression with 1 nN:

The contact area A between the bead and soma is given as area of the cap of the sphere with radius *r* at height *h*.

$$A = 2\pi\, rh$$

with *r* = 2.5 μm and *h* = 0.7 μm (indentation depth), the area *A* is:

$$A = 2\pi * (2.5) * (0.7) = 10.995 \approx 11.00\ \mu m^2$$

The pressure *P* is given as:

$$P = \frac{F}{A}$$

with *F* being the indentation force. The pressure *P* corresponding to the indentation force in the discussion section were calculated in the following way. For 1 nN, *P* is:

$$P = \frac{1 * 10^{-9}\ \mathrm{kg\ m\ s^{-2}}}{11.00 * 10^{-12}\ m^2} = 0.09 * 10^3 \mathrm{kg\ m^{-1}\ s^{-2}} \approx 0.09\ \mathrm{kPa}$$

Using this equation, we obtain a pressure value of ≈0.1 kPa for 1 nN of compression force**.** Applying force over a larger area reduces the pressure to sub-traumatic levels, thus minimizing the risk of neuronal injury as previously observed[3–7].

Supplementary References

1. Gaub, B. M. *et al.* Neurons differentiate magnitude and location of mechanical stimuli. *Proc. Natl. Acad. Sci. U. S. A.* **117**, 848–856 (2020).
2. Grevesse, T., Dabiri, B. E., Parker, K. K. & Gabriele, S. Opposite rheological properties of neuronal microcompartments predict axonal vulnerability in brain injury. *Sci. Rep.* **5**, 9475 (2015).
3. Zhou, C., Khalil, T. B. & King, A. I. *Shear Stress Distribution in the Porcine Brain due to Rotational Impact*. *J. Pass. Car.* vol. **103** 1697-1707 (1994).
4. Zhang, L., Yang, K. H. & King, A. I. A Proposed Injury Threshold for Mild Traumatic Brain Injury. *J. Biomech. Eng.* **126**, 226–236 (2004).
5. Hemphill, M. A., Dauth, S., Yu, C. J., Dabiri, B. E. & Parker, K. K. Traumatic brain injury and the neuronal microenvironment: A potential role for neuropathological mechanotransduction. *Neuron* **85**, 1177–1192 (2015).
6. Zhang, J., Pintar, F. A., Yoganandan, N., Gennarelli, T. A. & Son, S. F. Experimental Study of Blast-Induced Traumatic Brain Injury Using a Physical Head Model. *Stapp. Car Crash J.* **53**, 215–227 (2009).
7. Song, S. *et al.* A Wireless Intracranial Brain Deformation Sensing System for Blast-Induced Traumatic Brain Injury. *Sci. Rep.* **5**, 16959 (2015).
