# Supplementary material for: Mechanical stimulation and electrophysiological monitoring at subcellular resolution reveals differential mechanosensation of neurons within networks
Source: Nat Nanotechnol. 2024 Feb 20;19(6):825–33. doi: 10.1038/s41565-024-01609-1 (PMC11186759; doi:10.1038/s41565-024-01609-1)
Supplement: Supplementary file 2 — Reporting Summary [file 41565_2024_1609_MOESM2_ESM.pdf]

nature portfolio

Corresponding author(s): Prof. Dr. Daniel J. Müller
Prof. Andreas Hierlemann

Last updated by author(s): Jan 5, 2024

Reporting Summary

Nature Portfolio wishes to improve the reproducibility of the work that we publish. This form provides structure for consistency and transparency in reporting. For further information on Nature Portfolio policies, see our Editorial Policies and the Editorial Policy Checklist.

Statistics

For all statistical analyses, confirm that the following items are present in the figure legend, table legend, main text, or Methods section.

n/a	Confirmed	
☐	☒	The exact sample size (*n*) for each experimental group/condition, given as a discrete number and unit of measurement
☐	☒	A statement on whether measurements were taken from distinct samples or whether the same sample was measured repeatedly
☐	☒	The statistical test(s) used AND whether they are one- or two-sided<br>*Only common tests should be described solely by name; describe more complex techniques in the Methods section.*
☒	☐	A description of all covariates tested
☒	☐	A description of any assumptions or corrections, such as tests of normality and adjustment for multiple comparisons
☐	☒	A full description of the statistical parameters including central tendency (e.g. means) or other basic estimates (e.g. regression coefficient) AND variation (e.g. standard deviation) or associated estimates of uncertainty (e.g. confidence intervals)
☐	☒	For null hypothesis testing, the test statistic (e.g. *F*, *t*, *r*) with confidence intervals, effect sizes, degrees of freedom and *P* value noted<br>*Give P values as exact values whenever suitable.*
☒	☐	For Bayesian analysis, information on the choice of priors and Markov chain Monte Carlo settings
☒	☐	For hierarchical and complex designs, identification of the appropriate level for tests and full reporting of outcomes
☐	☒	Estimates of effect sizes (e.g. Cohen's *d*, Pearson's *r*), indicating how they were calculated

Our web collection on statistics for biologists contains articles on many of the points above.

Software and code

Policy information about availability of computer code

Data collection	All AFM measurements were collected using a Bruker (Version 9.1R3) and a JPK inbuilt data acquisition software was used (Versions 4.3.55 and 5.0135). For confocal laser scanning microscopy Zeiss (ZenBlue, version 3.5.093.00008) software was used. For upright fluorescence light microscopy Nikon NIS elements (Version BR 5.02.01) was used. All HD-MEA data was collected using MaxWell BioSystem MaxLab Live (Version 21.2.6 and 22.2.8)
Data analysis	Statistical tests were performed using Python (Version 3.6.10) with Scipy (Version 1.5.4). Stiffness measurements, cell height measurements, and indentation depth measurements were analyzed by using Bruker Nanoscope analysis software (Version 1.8), JPK data analysis software (JPK Instruments, version spm-4.3.55), and Python (Version 3.6.10). Images for confocal imaging were processed using ZEN Blue software (Zeiss, version 3.5.093.00008), Upright fluorescence microscopy data was analyzed with Nikon NIS elements (Version BR 5.02.01). Image processing was performed using Fiji (Version 2.1.0/1.52c).

For manuscripts utilizing custom algorithms or software that are central to the research but not yet described in published literature, software must be made available to editors and reviewers. We strongly encourage code deposition in a community repository (e.g. GitHub). See the Nature Portfolio guidelines for submitting code & software for further information.

Data

Policy information about availability of data

All manuscripts must include a data availability statement. This statement should provide the following information, where applicable:
- Accession codes, unique identifiers, or web links for publicly available datasets
- A description of any restrictions on data availability
- For clinical datasets or third party data, please ensure that the statement adheres to our policy

Source data are provided with this paper. A data availability statement is provided in the manuscript.

Human research participants

Policy information about studies involving human research participants and Sex and Gender in Research.

Reporting on sex and gender	Not human research participants were used in this study
Population characteristics	*Describe the covariate-relevant population characteristics of the human research participants (e.g. age, genotypic information, past and current diagnosis and treatment categories). If you filled out the behavioural & social sciences study design questions and have nothing to add here, write "See above."*
Recruitment	*Describe how participants were recruited. Outline any potential self-selection bias or other biases that may be present and how these are likely to impact results.*
Ethics oversight	*Identify the organization(s) that approved the study protocol.*

Note that full information on the approval of the study protocol must also be provided in the manuscript.

Field-specific reporting

Please select the one below that is the best fit for your research. If you are not sure, read the appropriate sections before making your selection.

☒ Life sciences ☐ Behavioural & social sciences ☐ Ecological, evolutionary & environmental sciences

For a reference copy of the document with all sections, see nature.com/documents/nr-reporting-summary-flat.pdf

Life sciences study design

All studies must disclose on these points even when the disclosure is negative.

Sample size	Sample size was not predetermined. The relevant individual cells used in generating data for each figure are provided in the legends of the figure. The experiments involving stiffness measurements on 50 individual neurons were performed at least on three different days. The mechanical stimulation and compression experiments on 300 individual neurons were performed on at least five different days.
Data exclusions	No analyzed data was excluded.
Replication	The single stiffness measurements were repeated on primary neurons prepared from two different animal dissections. The mechanical stimulation measurements were repeated on primary neurons prepared from five different animal dissections.
Randomization	Randomization was not relevant for this study.
Blinding	Blinding was not relevant to this study since measurements.

Reporting for specific materials, systems and methods

We require information from authors about some types of materials, experimental systems and methods used in many studies. Here, indicate whether each material, system or method listed is relevant to your study. If you are not sure if a list item applies to your research, read the appropriate section before selecting a response.

Materials & experimental systems

n/a	Involved in the study	
☒	☐	Antibodies
☒	☐	Eukaryotic cell lines
☒	☐	Palaeontology and archaeology
☐	☒	Animals and other organisms
☒	☐	Clinical data
☒	☐	Dual use research of concern

Methods

n/a	Involved in the study	
☒	☐	ChIP-seq
☒	☐	Flow cytometry
☒	☐	MRI-based neuroimaging

Animals and other research organisms

Policy information about studies involving animals; ARRIVE guidelines recommended for reporting animal research, and Sex and Gender in Research

Laboratory animals	E-18 Wistar rat embryos
Wild animals	Not applicable
Reporting on sex	Sex determination was not performed for the E-18 embryos used.
Field-collected samples	Not applicable
Ethics oversight	The experimental protocols involving animal tissue harvesting were approved by the veterinary office of the Canton Basel-Stadt according to Swiss federal laws on animal welfare and were carried out in accordance with the approved guidelines

Note that full information on the approval of the study protocol must also be provided in the manuscript.
